# Genetic variation at *MECOM*, *TERT*, *JAK2* and *HBS1L-MYB* predisposes to myeloproliferative neoplasms

**DOI:** 10.1038/ncomms7691

**Published:** 2015-04-07

**Authors:** William Tapper, Amy V. Jones, Robert Kralovics, Ashot S. Harutyunyan, Katerina Zoi, William Leung, Anna L. Godfrey, Paola Guglielmelli, Alison Callaway, Daniel Ward, Paula Aranaz, Helen E. White, Katherine Waghorn, Feng Lin, Andrew Chase, E. Joanna Baxter, Cathy Maclean, Jyoti Nangalia, Edwin Chen, Paul Evans, Michael Short, Andrew Jack, Louise Wallis, David Oscier, Andrew S. Duncombe, Anna Schuh, Adam J. Mead, Michael Griffiths, Joanne Ewing, Rosemary E. Gale, Susanne Schnittger, Torsten Haferlach, Frank Stegelmann, Konstanze Döhner, Harald Grallert, Konstantin Strauch, Toshiko Tanaka, Stefania Bandinelli, Andreas Giannopoulos, Lisa Pieri, Carmela Mannarelli, Heinz Gisslinger, Giovanni Barosi, Mario Cazzola, Andreas Reiter, Claire Harrison, Peter Campbell, Anthony R. Green, Alessandro Vannucchi, Nicholas C.P. Cross

**Affiliations:** 1 Faculty of Medicine, University of Southampton, Southampton SO16 6YD, UK; 2 Wessex Regional Genetics Laboratory, Salisbury District Hospital, Salisbury SP2 8BJ, UK; 3 CeMM Research Center for Molecular Medicine of the Austrian Academy of Sciences, Vienna 1090, Austria; 4 Haematology Research Laboratory, Biomedical Research Foundation, Academy of Athens, Athens 11527, Greece; 5 Department of Haematology, Addenbrooke’s Hospital, Cambridge CB2 0XY, UK; 6 Department of Haematology, University of Cambridge, Cambridge CB2 0XY, UK; 7 Laboratorio Congiunto MMPC, Department of Experimental and Clinical Medicine, University of Florence, Florence 50134, Italy; 8 Haematological Malignancy Diagnostic Service, St James's Institute of Oncology, Bexley Wing, St James's University Hospital, Leeds LS9 7TF, UK; 9 Department of Haematology, Royal Bournemouth Hospital, Bournemouth BH7 7DW, UK; 10 Department of Haematology, University Hospital Southampton, Southampton SO16 6YD, UK; 11 Oxford Biomedical Research Centre, Molecular Diagnostic Laboratory, Oxford University Hospitals NHS Trust, Oxford OX3 7LE, UK; 12 Haematopoietic Stem Cell Biology Laboratory, Weatherall Institute of Molecular Medicine, University of Oxford, Oxford OX3 9DS, UK; 13 School of Cancer Sciences, University of Birmingham,, Birmingham B15 2TT, UK; 14 West Midlands Regional Genetics Laboratory, Birmingham Women's NHS Foundation Trust, Birmingham B15 2TG, UK; 15 Birmingham Heartlands Hospital, Birmingham B9 5SS, UK; 16 Department of Haematology, UCL Cancer Institute, London WC1 E6BT, UK; 17 MLL Munich Leukaemia Laboratory, Munich 81377, Germany; 18 Department of Internal Medicine III, University Hospital of Ulm, Ulm 89081, Germany; 19 Institute of Epidemiology II, Research Unit of Molecular Epidemiology, Helmholtz Zentrum München, German Research Center for Environmental Health, Neuherberg 85764, Germany; 20 German Center for Diabetes Research, Neuherberg 85764, Germany; 21 Institute of Medical Informatics, Biometry and Epidemiology, Chair of Genetic Epidemiology, Ludwig-Maximilians-Universität, 80539 Munich, Germany; 22 Longitudinal Study Section, Translational Gerontology Branch, National Institute on Aging, Baltimore, Maryland 21224-6825, USA; 23 Geriatric Unit, Azienda Sanitaria Firenze (ASF), Florence 50122, Italy; 24 Medical University of Vienna, Department of Internal Medicine I, Division of Hematology and Blood Coagulation, Vienna 1090, Austria; 25 Center for the Study of Myelofibrosis, IRCCS Policlinico San Matteo Foundation, Pavia 27100, Italy; 26 Department of Molecular Medicine, University of Pavia, Pavia, Italy; 27 Department of Hematology Oncology, Fondazione IRCCS Policlinico San Matteo, Pavia 27100, Italy; 28 III. Medizinische Klinik, Universitätsmedizin Mannheim, Mannheim 68167, Germany; 29 Department of Haematology, Guy's and St Thomas' NHS Foundation Trust, Guy's Hospital, London SE1 9RT, UK; 30 Cancer Genome Project, Wellcome Trust Sanger Institute, Hinxton CB10 1SA, UK

## Abstract

Clonal proliferation in myeloproliferative neoplasms (MPN) is driven by somatic mutations in *JAK2*, *CALR* or *MPL*, but the contribution of inherited factors is poorly characterized. Using a three-stage genome-wide association study of 3,437 MPN cases and 10,083 controls, we identify two SNPs with genome-wide significance in *JAK2*^V617F^-negative MPN: rs12339666 (*JAK2;* meta-analysis *P*=1.27 × 10^−10^) and rs2201862 (*MECOM*; meta-analysis *P*=1.96 × 10^−9^). Two additional SNPs, rs2736100 (*TERT*) and rs9376092 (*HBS1L*/*MYB*), achieve genome-wide significance when including *JAK2*^V617F^-positive cases. rs9376092 has a stronger effect in *JAK2*^V617F^-negative cases with *CALR* and/or *MPL* mutations (Breslow–Day *P*=4.5 × 10^−7^), whereas in *JAK2*^V617F^-positive cases rs9376092 associates with essential thrombocythemia (ET) rather than polycythemia vera (allelic *χ*^2^ *P*=7.3 × 10^−7^). Reduced *MYB* expression, previously linked to development of an ET-like disease in model systems, associates with rs9376092 in normal myeloid cells. These findings demonstrate that multiple germline variants predispose to MPN and link constitutional differences in *MYB* expression to disease phenotype.

## Abstract (web summary)

Somatic mutations drive the clonal proliferation of myeloproliferative neoplasms. Here the authors conduct a genome-wide association study and identify germline variation at multiple loci associated with the development and disease phenotype of these cancers.

## Graphical abstract

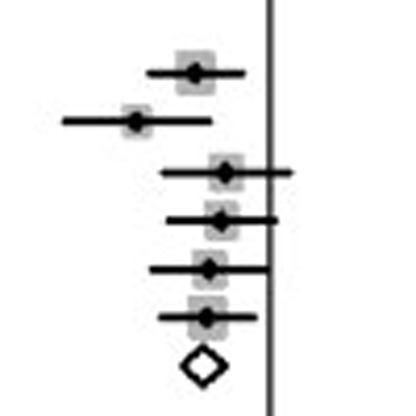

Myeloproliferative neoplasms (MPN) are a group of related haematological disorders that are characterized by an excess proliferation of one or more myeloid cell lineages and a tendency to transform to acute myeloid leukaemia^1^. MPNs are classified by which myeloid cell lineage(s) is predominantly expanded in the peripheral blood, namely erythrocytes in polycythemia vera (PV) and platelets in essential thrombocythemia (ET). Patients with primary myelofibrosis (PMF) display bone marrow fibrosis, megakaryocytic abnormalities, variable peripheral blood counts, extramedullary haematopoiesis and hepatosplenomegaly. MPNs arise as a consequence of clonal proliferation driven by at least one somatically acquired driver mutation. Of these, the most recurrent is *JAK2*^V617F^, which occurs in >95% of PV and 50–60% of ET and PMF cases. In patients without *JAK2* mutations, somatic mutations in *CALR* are the most common, occurring in well over 50% of *JAK2*^V617F^-negative ET and PMF. Other somatic mutations in MPN have been identified but they are less common and occur in fewer than 20% of cases. Most prominent of these from a phenotypic perspective are mutations that activate the thrombopoietin receptor (*MPL*), which are seen in 3–10% of ET and PMF. In the great majority of cases, mutations in *JAK2*, *CALR* and *MPL* are mutually exclusive, although occasional cases may test positive for mutations in more than one of these genes. Patients with ET and PMF that test negative for *JAK2*, *CALR* and *MPL* mutations are typically referred to as ‘triple negative’^2^,^3^.

Although it is accepted that acquisition of these mutations drives clonal proliferation in MPNs, less is known about what factors influence the development, phenotype and severity of disease. Evidence from epidemiological^4^ and familial studies^5^,^6^ strongly suggest that common, low penetrance factors present in the general population contribute to the risk of developing MPN and potentially such factors may also contribute to the phenotypic pleiotrophy observed in these disorders. We previously characterized a major predisposition locus at the *JAK2* gene, which is associated with the acquisition of V617F mutations^7^,^8^. This specific *JAK2* haplotype, called ‘46/1’ or ‘GGCC’ strongly predisposes to *JAK2*^V617F^-positive disease (OR=3.7; Fisher’s exact test *P*<10^−20^). The 46/1 haplotype is also weakly associated with *JAK2* exon 12 positive PV^9^, MPN with *MPL* W515 mutations and those that lack *JAK2* or *MPL* mutations^10^. More recently, a common germline variant was identified in *TERT*, which predisposes equally to PV (OR=2.3, allelic *χ*^2^ *P*=1.1 × 10^−5^), ET (OR=2.3, allelic *χ*^2^ *P*=2.87 × 10^−3^) and PMF (OR=2.4, allelic *χ*^2^ *P*=2.46 × 10^−2^) in the Icelandic population^11^.

Overall, the 46/1 haplotype has been estimated to account for ∼50% of the population attributable risk (PAR) of developing an MPN in European populations, but it only explains a minor component of *JAK2*^V617F^-negative disease^7^. The variant in *TERT* is also estimated to have a PAR of 58% in Icelandics and contributes to *JAK2*^V617F^ positive and negative disease. Since the bulk of the population-level predisposition to *JAK2*^V617F^ positive MPN is likely to be accounted for by variation at *JAK2* and *TERT*, the initial aim of this study is to identify additional germline variants that predispose to *JAK2*^V617F^-negative ET or PMF by performing a genome-wide association study (GWAS).

We identify two single-nucleotide polymorphisms (SNPs), rs12339666 within *JAK2* and rs2201862, 153 kb downstream of *MECOM*, which associate with *JAK2*^V617F^-negative disease, and two additional SNPs, rs2736100 in *TERT* and rs9376092 between *HBS1L* and *MYB*, when including *JAK2*^V617F^-positive cases. The SNP between *HBS1L* and *MYB,* rs9376092, has a stronger effect in *JAK2*^V617F^-negative cases with somatic *CALR* and/or *MPL* mutations and predisposes to essential thrombocythemia in *JAK2*^V617F^-positive cases. Finally, we demonstrate that the candidate risk allele at rs9376092 is associated with reduced *MYB* expression in normal myeloid cells.

## Results

### Genome-wide association study

**Figure 1:**
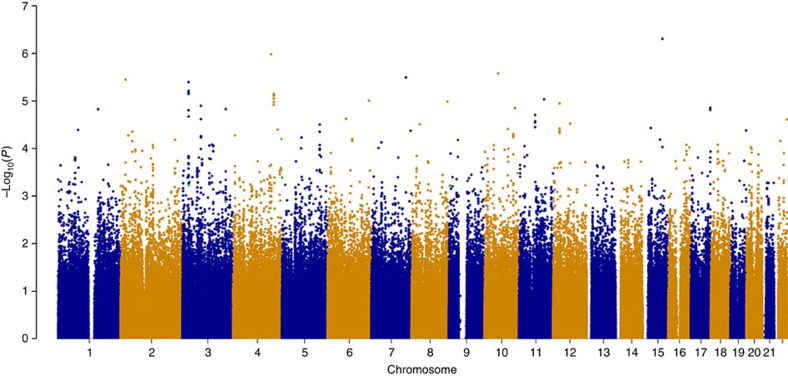
Genome-wide association of JAK2^V617F^-negative myeloproliferative neoplasms. This Manhattan plot shows the results of the stage 1 GWAS for all autosomes. Results are plotted as –log10 of the allelic *χ*^2^ *P* value. At this stage, no SNPs met the stringent genome-wide significance level of *P* value ≤10^−8^. The three SNPs that attained genome-wide significance and two additional SNPs with moderate association following meta-analysis are highlighted in green, on chromosome 3 (rs2201862 and rs4858647), chromosome 5 (rs2736100), chromosome 6 (rs9376092) and chromosome 9 (rs12339666). This plot was produced using the qqman R package.

Following quality control there were 524 *JAK2*^V617F^-negative MPN cases, 2,674 controls and 2,098,039 SNPs (642,633 observed and 1,455,406 imputed) for analysis at stage-1 (Supplementary Fig. 1). This cohort is estimated to have 80% power for detecting common SNPs (minor allele frequency (MAF)=0.3) with relative risks of 1.25 and rare SNPs (MAF=0.05) with relative risks (RR) of 1.5 (ref. 12). The QQ plot demonstrated good agreement between observed and expected *P* values (genomic inflation factor, *λ*=1) until the tail of the distribution where SNPs with *P* values <10^−4^ deviated from the null distribution (Supplementary Fig. 2). Systematic biases between cases and controls such as population stratification or somatic mutations in the MPN cases are therefore unlikely to contribute to the significance of SNPs. Figure 1 shows a Manhattan plot of these results. Following our selection criteria (see Methods), 203 SNPs from 173 genomic regions with allelic *χ*^2^ *P* values ≤0.02 (Supplementary Table 1) were chosen for assessment in the three-stage two cohorts.

### Replication and validation of candidate SNPs

For replication at stage-2, 196 of the 203 selected SNPs were successfully genotyped in 658 *JAK2*^V617F^-negative MPN cases and 1,196 controls (Supplementary Table 1). A further three SNPs were removed due to significant deviation from Hardy–Weinberg equilibrium (HWE; exact test *P*-value ≤0.001 in controls) leaving 193 SNPs for analysis. Additional cases from Germany (*n*=187) and Austria (*n*=99) and controls from the BBC (*n*=2706) and from Bavaria (*n*=1805), all of which had been previously genotyped, were also used. These cases and controls were analysed as three separate cohorts consisting of samples from the United Kingdom (stage-2.1), Germany and Austria (stage-2.2), and Greece (stage-2.3). After testing for allelic association and using a fixed effects meta-analysis to combine evidence from stages 1 and 2, five SNPs were identified with combined *P* values ≤0.001 and significant replication in at least one of the three replication cohorts with effects in the same direction as the stage-1 analysis (Supplementary Table 1). The uncombined replication *P*-values for allelic *χ*^2^ tests ranged from 0.025 to 0.00025. One of these SNPs (rs12339666) is located within the *JAK2* gene and was not considered further because of its consistency with previous findings, showing that variants tagging the *JAK2* 46/1 haplotype are associated with *JAK2*^V617F^-negative MPN^10^.

A total of four SNPs with replication at stage-2 were therefore selected for further analysis (stage-3) in two independent cohorts consisting of 328 cases and 2,800 controls from the United Kingdom (stage-3.1) and 637 cases and 160,8 controls from Italy (stage-3.2, Supplementary Table 1). Of the four SNPs tested, all of which were successfully genotyped and passed quality criteria, three (rs9376092, rs4858647 and rs2201862) had allelic *χ*^2^ *P* values <0.05 and effects in the same direction as the stage-1 analysis and remained significant when considering the false discovery rate^13^ to correct for the number of SNPs tested (Supplementary Table 1). To further validate our findings, the four SNPs were genotyped in 406 reactive cases from the United Kingdom, which mimic MPNs and were thus considered as negative controls. Comparison to 2,800 controls from the United Kingdom (stage 3.3) indicated that none of the SNPs were significantly associated with the risk of developing a reactive phenotype (Supplementary Table 1), thus supporting the notion that they are specifically associated with clonal MPN.

**Figure 2:**
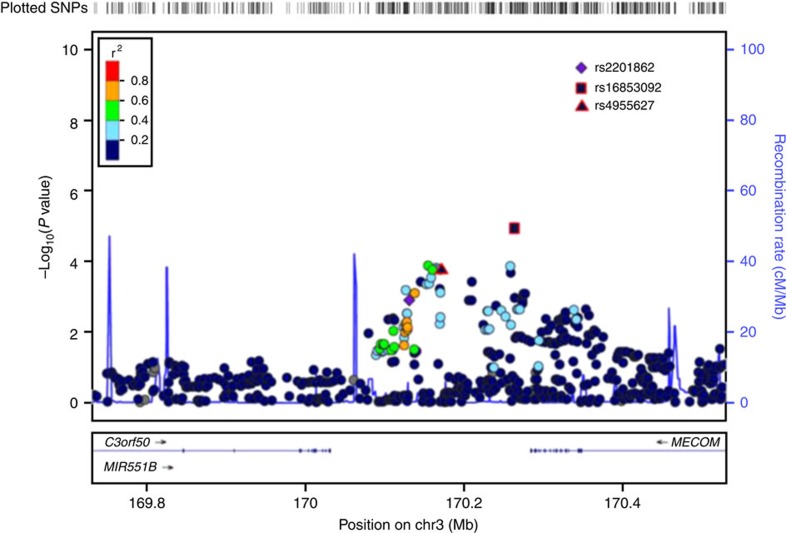
A plot of the stage 1 GWAs for the most significant novel SNP (rs2201862) identified by meta-analysis. This plot shows the stage 1 results (*n*=524 JAK2^V617F^-negative cases versus *n*=2674 controls) in a 400-kb region, which flanks the most significant SNP identified by meta-analysis (rs2201862). SNPs are plotted as the −log10 of the allelic *χ*^2^ *P* value. The amount of LD between rs220186 and all other SNPs is indicated by the colour of the data points. The two SNPs with red borders (rs16853092 and rs4955627) were also genotyped and replicate at stage 2 but were not taken forward to stage 3 because rs2201862 was more significant at stage 2. The plot was produced using LocusZoom.

**Figure 3:**
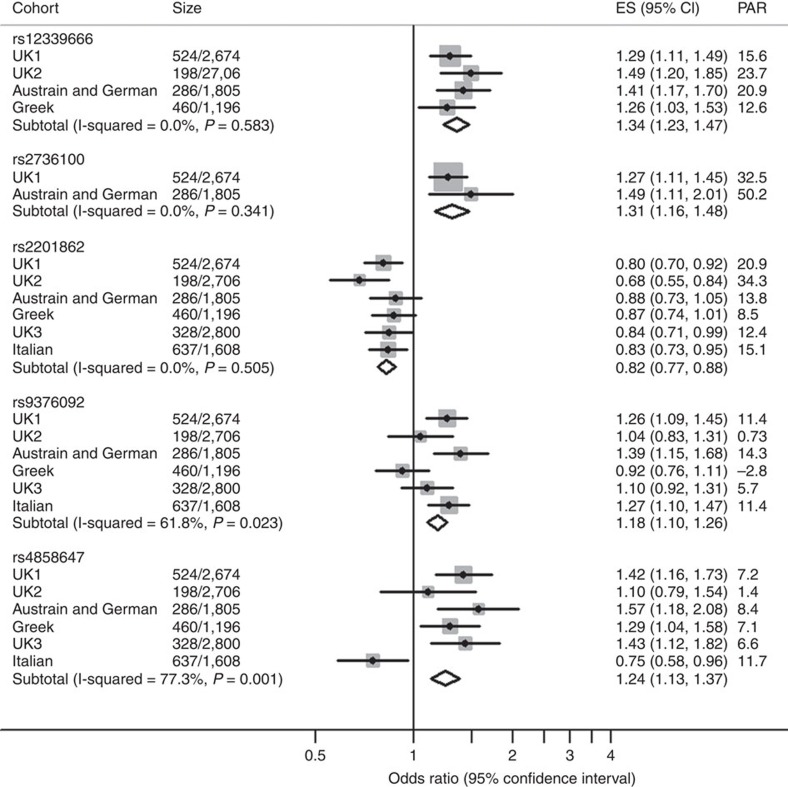
Forest plots and meta-analysis of genotype data for the five most significant associations. Forest plots showing the odds ratios (OR=ES), 95% confidence intervals (CI) and population attributable risk (PAR) of SNPs with or approaching a genome-wide level of significance in each cohort and in the combined analysis. The SNP subtotals show the OR and CI for a fixed-effects meta-analysis and use I-squared to assess heterogeneity in effect sizes between cohorts.

Following meta-analysis of the six cohorts using fixed-effect sizes, one novel SNP was identified with a genome-wide level of significance (rs2201862; meta-analysis *P*=1.96 × 10^−9^), an effect in the same direction as the discovery data, and without significant heterogeneity between cohorts (Table 1, Figs 2 and 3). Furthermore, rs2201862 had evidence for replication (*P*≤0.05 for allelic *χ*^2^) in three of the five follow-up cohorts and two unlinked SNPs (*r*^2^<0.5) that are within 100 kb are also significant (*P*≤0.05 for allelic *χ*^2^-test) in one of the three replication cohorts that were tested for these SNPs. Rs2201862 is located at chromosome 3q26 ∼153 kb downstream of *MECOM* and 99 kb downstream of a pseudogene *EGFEM1P*. In addition, two other SNPs had moderate evidence of association (rs9376092 meta-analysis *P*=5.27 × 10^−6^ and rs4858647 meta-analysis *P*=1.39 × 10^−5^) and effects in the same direction as the discovery data (Table 1, Fig. 2). However, for both these SNPs Cochran’s *Q* statistic and the *I*^2^ statistic estimated significant heterogeneity between cohorts. Rs9376092 is located at 6q23, 51 kb upstream of *HBS1L* and 76 kb upstream of *MYB*. Rs4858647 is an intergenic SNP at 3p24, 255 kb upstream of *THRB* and 425 kb upstream of *RARB*.

### Association with variation at *TERT*

Following the discovery of an association between rs2736100 in *TERT* at 5p15 and MPN^11^, we re-examined our data for evidence of this relationship. In our original analysis, rs2736100 was not tested at stage-1 due to its significant deviation from HWE in the pooled cohort of controls from WTCCC2 (NBS+BBC *P*=0.00096 for HWE exact test). However, when controls from the NBS and BBC cohorts were assessed separately the deviation from HWE did not exceed the QC threshold of *P*≤0.001 (NBS *P*=0.01, BBC *P*=0.03 for HWE exact test). The stage 1 data were therefore reanalysed using the unpooled assessment of HWE, which identified rs2736100 as the only SNP with a *P* value ≤0.01 (*P*=0.0004, OR=1.27 for allelic *χ*^2^) in a 242-kb region, which flanks the *TERT* locus by 100 kb. This association was replicated in the Austrian cohort (*P*=0.008, OR=1.49 for allelic *χ*^2^), and a meta-analysis of the two cohorts was used to estimate the final significance and effect size (Table 1: meta-analysis *P*=1.73 × 10^−5^, OR=1.31). In both cohorts, rs2736100 was not correlated with any other SNPs (*r*^2^>0.15), which may account for the lack of supporting SNPs within the *TERT* locus. Other *JAK2*^V617F^-negative cohorts were not tested for rs2736100.

### Associations with *CALR* and *MPL* mutations

To investigate whether the SNPs we identified had similar effects in cases with and without somatic mutations of *CALR* and/or *MPL*, the 6 *JAK2*^V617F^-negative cohorts were stratified according to the presence or absence of these mutations and compared using a Breslow–Day test (Table 2B). This analysis determined that the effects of rs12339666 and rs9376092 were significantly lower in cases without *CALR* and/or *MPL* mutations, while rs2201862, rs4858647 and rs2736100 had effects that were similar in cases with and without these mutations. Of these, rs9376092 (*HBS1L-MYB*) was particularly prominent (Breslow–Day *P*=4.5 × 10^−7^; Table 2B).

To investigate the possibility that germline variation at *CALR* might contribute to the acquisition of somatic *CALR* mutations, a 206-kb region that flanks the *CALR* locus by 100 kb and contains 11 SNPs was investigated in the stage 1 data as a whole and in the subset of cases with *CALR* mutations. No significant SNPs were identified in this region in either data set (Supplementary Fig. 3). However, the closest SNPs to *CALR* in this region are rs11667458, which is 29,869 bp downstream, and rs10403210, which is 48,335 bp upstream, and neither of these are in linkage disequilibrium (LD) with any SNPs in or close to *CALR*. Additional studies are therefore required to determine definitively whether germline variation at *CALR* influences the acquisition of somatic *CALR* mutations.

### Associations with *JAK2* ^V617F^-positive MPN

To determine whether any of the SNPs were associated exclusively with *JAK2*^V617F^-negative MPN, two cohorts of *JAK2*^V617F^-positive cases (PV, *n*=505; ET, *n*=499) and 2,706 controls from the United Kingdom were analysed (stage-3.4 and stage 3.5). After testing for allelic association and using the false discovery rate^13^ to correct for the five SNPs tested, all SNPs except rs4858647 retained significance in at least one of the two cohorts and had effects in the same direction as the previous analyses (Supplementary Table 1). On comparison of *JAK2*^V617F^ positive and negative MPN, the Breslow–Day test identified two SNPs that had stronger effects in *JAK2*^V617F^-positive cases: rs12339666 (Breslow–Day *P*=2.8 × 10^−18^), which tags the *JAK2* 46/1 haplotype, and rs2736100 (*TERT*; Breslow–Day *P*=0.0026). Rs4858647 (*THRB-RARB*) had a stronger effect in *JAK2*^V617F^-negative cases (Breslow–Day *P*=0.015), and no difference was seen for the remaining SNPs rs2201862 (*MECOM*) and rs9376092 (*HBS1L-MYB*) (Table 2B).

Comparison of effect sizes between *JAK2*^V617F^-positive ET versus controls and *JAK2*^V617F^-positive PV versus controls revealed significantly larger effect for rs12339666 in PV (*JAK2*; Breslow–Day *P* =2.452 × 10^−11^) and rs9376092 in ET (*HBS1L-MYB*; Breslow–Day *P*=0.0004; Table 2B). Meta-analysis of all *JAK2*^V617F^-negative and *JAK2*^V617F^-positive cases (Table 1) identified three SNPs that achieved genome-wide significance: rs12339666 (*JAK2*), rs2736100 (*TERT*) and rs2201862 (*MECOM*). Rs9376092 (*HBS1L-MYB*) also achieved genome-wide significance in meta-analysis of all *JAK2*^V617F^-negative and *JAK2*^V617F^-positive ET and MF cases (Table 1).

### Population attributable risks

According to the genotypic odds ratio and population frequency, rs2736100 is estimated to account for the largest proportion of the population attributable risk in *JAK2*^V617F^-negative cases (PAR=34.9%) followed by rs12339666, which tags the 46/1 haplotype (PAR=17.4% compared with 11.8% for the tightly linked SNP rs12340895 in *JAK2*^V617F^ and *MPL* mutation negative cases from our previous study^10^). The SNP in close proximity to *MECOM*, rs2201862, accounts for 16.5% of the PAR (Table 1). On the basis of a multiplicative model without interaction, these three factors are estimated to have a combined PAR of 55%.

### Variation at rs9376092 correlates with *MYB* expression

The candidate MPN risk SNP rs9376092 maps to the *HBS1L* and *MYB* intergenic polymorphic region (HMIP), an area that has been linked genetically to a number of hematological traits in healthy individuals, including platelet number^14^,^15^. Independent functional analysis has shown that knockdown mice expressing low levels of Myb develop a transplantable ET-like disease^16^. We therefore tested whether variation at rs9376092 was linked to differences in expression of *MYB* in healthy individuals. We found that the risk allele was indeed associated with a 5.8- to9.7-fold reduction of *MYB* expression in BFU-E and CFU-GM. (Supplementary Table 2, Supplementary Fig. 4a). The risk allele was also associated with reduced *HBS1L* expression, but this was less marked (1.9- to 2.4-fold). In addition, we interrogated our previously published gene expression data set derived from haemopoietic colonies of MPN cases^17^. *MYB* expression in ET was significantly lower in *JAK2*^V617F^-heterozygous BFU-E colonies compared with *JAK2* wild-type colony cells from the same patient (Mann–Whitney test *P*=0.0009; q=0.01), but this difference was not seen in colonies from PV cases. For *JAK2*, no difference was seen in either ET or PV (Supplementary Fig. 4b).

The other risk variants were not amenable to similar analysis because the associated candidate genes were not sufficiently expressed in PB granulocytes or colonies. Instead, we investigated the relationship between risk alleles and the expression of flanking genes using GENEVAR and the eQTL database hosted by the University of Chicago^18^. There were no correlations of any sentinel SNP or SNP in high LD (*r*^2^>0.8) with gene expression measured in lymphoblastoid cell lines.

### Association with clinical features in the PT-1 trial

To determine whether variants that predispose to the development of MPN confer a functional effect on particular aspects of MPN disease features, we correlated genotypes of the top associations with clinical and laboratory measurements and outcome recorded for *JAK2*^V617F^-negative patients recruited into the PT-1 trial^19^ (Supplementary Table 3; absence of correlations with the *JAK2* 46/1 haplotype has been reported previously^10^). There were no striking associations with any aspect of laboratory and clinical features at diagnosis, but in events after trial entry, there were weak associations of rs3737304 and rs9376092 with transformation to myelofibrosis, and venous thromboembolism in the year before diagnosis, respectively. There was also a weak association of rs4858647 on haemoglobin levels recorded in response to therapy. None of these associations, however, retained significance after correction for multiple testing.

## Discussion

We focused initially on identifying common inherited genetic factors that predispose to *JAK2*^V617F^-negative MPN. We analysed DNA extracted from peripheral blood leukocytes, which would be expected to include a variable proportion of clonal and non-clonal cells. We considered the possibility that clonal somatic changes might affect the analysis; however, in line with previous array and exome-sequencing studies^3^,^20^ we found that MPN genomes are relatively simple compared to other cancers with only 8% (42/524) of cases showing likely somatic copy-number changes or acquired uniparental disomy (Supplementary Fig. 5). These regions of gross alteration do not correspond to the location of the risk factors we identified and exclusion of these cases has minimal effect on the overall results (Supplementary Table 6). Although we cannot completely exclude the possibility that recurrent clonal changes below the resolution of array analysis might have influenced our findings, SNPs in such regions would generally be excluded by our rigorous QC criteria due to a high percentage of missing genotypes, poor genotyping quality or deviation from HWE. Notably, none of the associated SNPs deviated from HWE at an Exact test *P* value ≤0.05, which suggests that clonal changes have not contributed to their significance (Methods and Supplementary Table 7).

Meta-analysis of six *JAK2*^V617F^-negative cohorts from our three-stage GWAS identified two SNPs with genome-wide significance and three that approached this level of significance. The most significant SNPs are rs12339666, which tags the *JAK2* 46/1 haplotype, and rs2201862, which is in a non-coding region proximal to *MECOM*. Of the three SNPs with moderate association, two are within or close to genes of potential functional relevance to MPN: the intergenic region between *HBS1L* and *MYB* and an intronic SNP in *TERT*, which has recently been associated with MPN in an independent study^11^, as well as several other malignancies. The remaining SNP (rs4858647) was mapped to a gene desert on chromosome 3p24. Although the *TERT* SNP rs2736100 did not achieve genome-wide significance (Table 1), it should be noted that we only focused on this polymorphism retrospectively and thus the number of cases analysed was significantly fewer than for the other variants. When the *JAK2*^V617F^-positive cohort was included in the analysis, rs2736100 also achieved genome-wide significance (Table 1).

To our knowledge, only one other polymorphism has been reported in the literature to be associated with the development of MPN: rs6198 in the 3′ UTR of *NR3C1*, which encodes the glucocorticoid receptor. On the basis of a very small number of cases, rs6198 was suggested to be associated with PV and MF, but not ET^21^. An association with MF, however, was confirmed in a subsequent larger study^22^. This SNP is not included on the SNP 6 platform and thus was not formally evaluated in our study. However, we did not see any suggestion of an association with other SNPs at *NR3C1* in our stage-1 cohort, which consisted of cases with ET.

The *JAK2* and *TERT* SNPs had effects that were stronger in *JAK2*^V617F^-positive cases, whereas rs4858647 (*THRB-RARB*) was stronger in *JAK2*^V617F^-negative cases. Within *JAK2*^V617F^-negative cases, the effect of rs9376092 (*HBS1L-MYB)* was markedly stronger in cases with *CALR* and/or *MPL* mutations compared with those that were triple negative. Overall, the three principal loci (*JAK2/TERT/MECOM*) are estimated to have a combined PAR of 51% for *JAK2*^V617F^-negative MPN. Fine mapping may uncover some of the missing heritability, either by identifying causal variants that are more strongly associated or by identifying additional signals at these loci. Nevertheless, it is likely that there are additional loci with equivalent or lesser effects that predispose to MPN, which could be detected by larger follow-up studies. Our preliminary analysis of the impact of the new associations on clinical and biological parameters recorded for the PT-1 trial have suggested only weak effects at best, but it remains to be determined whether integration of these genetic variants into risk models, together with that of somatically acquired mutations, may enhance risk profiling and be clinically useful. Nevertheless, identification of genetic predisposition loci provides pointers towards novel biological mechanisms that underlie MPN development.

Some of the associations we identified are close to loci with established credentials of relevance to MPN. The strongest novel association was rs2201862, 153Kb downstream of *MECOM* (*MDS1* and *EVI1* complex locus), which has been implicated in megakaryopoiesis by several lines of evidence. First, a number of alternatively spliced mRNAs are transcribed from this locus and one of these (*EVI1*) is overexpressed in myeloid malignancies with chromosome rearrangements, usually an inv(3)(q21q26) or t(3;3)(q21;q26) that reposition a *GATA2* enhancer at 3q21 downstream of *MECOM* at 3q36 (ref. 23). Affected cases typically display abnormal megakaryocyte morphology and elevated platelet counts^24^. Second, overexpression of *EVI1* blocks terminal differentiation of bone marrow progenitor cells to granulocytes^25^ but promotes megakaryocyte differentiation in UT-7/GM cells^26^. Third, the genetic basis of most cases of TAR (thrombocytopenia with absent radii) syndrome is a null allele of *RBM8A* coupled with a rare polymorphism that creates an EVI1 binding site at the *RBM8A* promoter, resulting in greatly reduced levels of *RMB8A* expression^27^. Owing to a highly restricted expression pattern in haematopoietic progenitors, we were unable to determine whether rs2201862 influences *MECOM* expression; however, mining of publicly available databases indicated that this SNP, which is in a region of very low LD, co-localizes with a DNAse1-hypersensitive site in the embryonic stem cell line h7-hESC, suggesting it may correspond to a regulatory element with enhancer-like activity.

Although rs9376092 did not reach genome-wide significance in *JAK2*^V617F^-negative cases alone, other data points to its importance as a risk allele in MPN. This SNP maps to a well-characterized region on chromosome 6q23 located 51 Kb centromeric of *HBS1L* and 75Kb telomeric of *MYB. MYB* encodes a transcription factor present at high levels in immature progenitors that plays an important role in progression through key stages of haemopoiesis^28^. *HBS1L* encodes a GTP-binding elongation factor that is involved in the regulation of a variety of cellular processes, such as protein synthesis, cytoskeletal trafficking and signal transduction^29^. In healthy individuals, diverse SNPs in the HMIP region between *HBS1L* and *MYB* were initially found to be associated with hereditary persistence of fetal haemoglobin^30^. Subsequent studies identified that HMIP SNPs are associated with normal variation in platelet, monocyte and erythrocyte counts as well as erythroid phenotype^15^,^31^,^32^,^33^,^34^. The SNP identified by our GWAS, rs9376092, is also located in the HMIP region and is in strong linkage disequilibrium with other variants that tag this block. Recently, HMIP variation has also been linked to the risk of developing Hodgkin’s lymphoma^35^.

Several studies have focused on the definition of HMIP as an erythroid distal regulatory region. HMIP shows DNase I hypersensitivity, GATA binding, RNA polymerase II interaction and marked histone acetylation^36^,^37^,^38^. In erythroid progenitor cells grown in liquid culture, HMIP variation correlated with both *HBS1L* and *MYB* expression, but the effect was more pronounced for *HBS1L* and only *HBS1L* expression correlated with elevated levels of fetal haemoglobin^39^. However, the multiple genetic associations described above indicate that HMIP influences other myeloid lineages, suggesting that it may harbour multiple tissue-specific enhancer elements. Indeed, we found that allelic variation at rs9376092 was associated with differences in expression of both flanking genes in healthy individuals but most markedly with *MYB*, with the risk allele linked to reduced *MYB* expression. We also found that *MYB* expression is lower in *JAK2*^V617F^ mutant BFU-E from ET patients compared with wild-type BFU-E. These observations are in agreement with previous findings that genetic knockdown of Myb in mice induces a transplantable MPN that closely resembles human ET^16^ and that *MYB* knockdown in CD34+ human bone marrow cells promotes megakaryocyte development^40^. Thus, our findings link a candidate risk SNP to lower levels of *MYB* expression, which has been shown to promote an ET-like disease in mice and megakaryocytic development in humans. Strikingly, in *JAK2*^V617F^-positive cases we find that variation at rs9376092 is strongly linked to an ET phenotype. Direct comparison of SNP frequencies between *JAK2*^V617F^-positive ET and *JAK2*^V617F^-positive PV indicates that the rs9376092 C allele is more common in ET (allelic *χ*^2^ *P*=7.5 × 10^−7^; Table 3), whereas comparison with controls indicates that this SNP specifically increases the risk of ET (allelic *χ*^2^ *P*=2.6 × 10^−6^; Table 2B), and there is a trend towards a reduction in the risk of developing PV (allelic *χ*^2^ *P*=0.068) since the effects are in the opposite direction for these two subtypes (Table 2B). It is well established that homozygous *JAK2*^V617F^-positive clones are much more prominent in PV than ET^41^, an observation that is consistent with the suggestion that elevated JAK2 signalling promotes an erythroid phenotype. The finding that leukocyte counts in murine *JAK2*^V617F^ retroviral-mediated transfection/transplantation models differ in a strain-dependent manner was the first suggestion that host genetic factors may influence the disease phenotype^42^. Here, we have identified *HBS1L-MYB* as a locus outside *JAK2* that influences the phenotype of human MPN.

In conclusion, our study indicates that genetic variation at multiple loci contributes to the risk of developing a MPN. The functional basis for these associations is largely unknown, but we have linked a candidate risk SNP at 6q26 to lower levels of *MYB* expression and an ET phenotype. Further investigation will be needed to understand how genetic variation at *JAK2*, *MECOM*, *TERT* and other loci predispose to MPN.

## Methods

### Stage-1 discovery data set

Before quality control, the stage-1 cohort consisted of 571 cases of *JAK2*^V617F^-negative MPNs from the United Kingdom (referred to as UK1), which comprise 556 with ET and 15 with PMF. Of these, 392 were sourced from the UK Primary Thrombocythaemia 1 (PT-1) study, which included newly diagnosed and previously treated patients, aged 18 years or over, who met the Polycythemia Vera Study Group (PVSG) criteria for ET^19^. One hundred and one are from the NCRI MPN sample bank at Addenbrookes Hospital, Cambridge. The remaining cases (*n*=78) were recruited from the NCRI MPN Study Group members following clinical and morphological review to identify *bona fide* MPN cases. For stage-1 controls, the National Blood Service (NBS) cohort from the Wellcome Trust Case Control Consortium (WTCCC2; *n*=2674; www.wtccc.org.uk) was used.

### Replication cohorts

Five independent cohorts of *JAK2*^V617F^-negative MPN who met the Polycythemia Vera Study Group or World Health Organization criteria for ET or MF were used to validate significant SNPs from stage-1 (Supplementary Table 1). These cohorts consisted of 198 cases from the United Kingdom and 2,706 controls from the WTCCC2 1958 British Birth Cohort (BBC, Stage-2.1), 286 cases from a combination of Austrian^43^ (*n*=99) and German cases (*n*=187) and 1,805 Bavarian controls from the German Cooperative Health Research in the Region of Augsburg cohort (KORA F4; Stage-2.2)^44^, 460 Greek cases and 1,196 locally recruited Greek controls (Stage 2.3), 328 cases from the United Kingdom and 2,800 controls from a combination of controls from BBC (*n*=2706) and the United Kingdom (*n*=94) (Stage-3.1), and 637 Italian cases and 1,608 Italian controls (Stage-3.2) from a combination of controls from the Invecchiare in Chianti study (InCHIANTI; *n*=1210)^45^, and a series of locally recruited Italian controls (*n*=398). To further validate our findings, 406 UK cases with elevated levels of platelets and/or erythrocytes and a reactive cause were identified. These reactive cases mimic MPN but are non-clonal and were used as a negative control by comparison with 2,706 controls from the BBC (Stage-3.3).

To determine whether SNPs with significant replication in one or more of the five follow-up cohorts are associated exclusively with *JAK2*^V617F^-negative MPN, two independent cohorts of consisting of 556 ET and PMF *JAK2*^V617F^-positive MPN cases and 556 PV *JAK2*^V617F^-positive MPN cases from the United Kingdom were genotyped and compared with 2,706 controls from the BBC (Stage-3.4 and 3.5 Supplementary Table 1). The study was approved by the following Internal Review Boards and/or ethics committees: National Research Ethics Service (UK) Committee South West; London Multicentre Research Ethics Committee; Cambridgeshire4 Research Ethics Committee; Trent Research Ethics Committee; Ethics Committee of the Biomedical Research Foundation of the Academy of Athens; Comitato Etico, Azienda Ospedaliero-Universitaria Careggi, Firenze; Comitato di Bioetica, Fondazione IRCCS Policlinico San Matteo, Pavia; Ethic committee of the Bavarian State Chamber of Physicians; Ethic committee of the University of Ulm; Medizinische Ethikkommission II der Medizinischen Fakultät Mannheim; Ethikkommission der Medizinischen Universität Wienand informed consent was obtained from participants according to the Declaration of Helsinki.

### Genotyping

The stage 1 cases were genotyped by AROS Applied Biotechnology (Aarhus, Denmark) using Affymetrix SNP6 arrays. To reduce genotyping errors and ensure harmonization of genotype calls across plates, the raw data (cel files) were pooled and the Birdseed v2 algorithm in Affymetrix’s genotyping console was used to determine genotypes. The stage 1 controls were previously genotyped by the WTCCC using Affymetrix SNP6 arrays and the CHIAMO algorithm to determine genotypes. Excluding the WTCCC2 controls, KORA controls and Austrian cases, the replication cohorts were genotyped by LGC Genomics (formerly KBiosciences, Hoddesdon, UK). In the initial validation round (Stage 2), 203 SNPs were genotyped and five of these SNPs were selected for genotyping in the second validation round (Stage 3). The Austrian cases, Bavarian controls and WTCCC2 controls were previously genotyped using Affymetrix SNP6 arrays. Stage-3.4 and stage-3.5 *JAK2*^V617F^-positive MPN cases from the United Kingdom were genotyped using the Illumina Human OmniExpressExome v1.2 platform by Gen-Probe Life Sciences Ltd (Wythenshawe, UK).

### Quality control for genotyping

Quality control of the stage-1 genotypes involved the removal of samples with poor resolution of SNP clusters (*n*=12 samples with contrast QC <0.4), SNPs with MAF <0.01, SNPs without observations for all three genotypes, SNPs and samples with >10% missing genotypes. A number of measures were used to control for poor genotyping: first, SNPs with significant deviations from Hardy–Weinberg equilibrium (HWE) in control samples (Exact test *P*-value ≤0.001) were removed. Since the cases and controls at stage-1 were not genotyped at the same time, we also removed SNPs with extreme deviation from HWE in cases (Exact test *P*-value ≤1 × 10^−10^) and SNPs with an average genotyping confidence call over all cases that were >2 s.d. values worse than the mean over all SNPs to control for potential genotyping errors in cases. To further control for poor genotyping, SNPs with allele frequencies that were significantly different between the two WTCCC2 control cohorts (NBS and BBC) and Caucasian controls from HapMap were also removed. Finally, SNPs located on non-autosomal chromosomes were removed and CNVs were not considered. A total of 266,988 SNPs were removed from the discovery cohort during these QC steps leaving observed genotypes at 642,633 SNPs for analysis.

Pairwise identity by state scores (IBS) were used to test the stage-1 samples for cryptic relatedness and nine individuals with IBS scores >86% were excluded from the stage-1 samples. These IBS scores were also used to perform a multi-dimensional scaling analysis (MDS) to identify ethnic outliers when referenced against the HapMap populations. On this basis, 26 of the stage-1 samples whose genotypes did not concur with a European ancestry were removed. The remaining cases clustered tightly with the WTCCC2 controls in the MDS plot, which demonstrates that our cases and controls are ethnically compatible (Supplementary Fig. 6). After these quality control procedures 524 cases (from 571 genotyped) and 2,674 controls remained for analysis in the discovery cohort. Of the 556 ET and PMF *JAK2*^V617F^-positive MPN cases (stage-3.4) and 556 PV *JAK2*^V617F^-positive MPN cases (stage-3.5), 499 and 505 remained for analysis. All quality control steps were performed using PLINK^46^.

To increase the resolution of the stage 1 data, additional SNPs were imputed using MACH 1.0 (http://www.sph.umich.edu/csg/abecasis/MACH/index.htm) and reference data from HapMap phase 2 in the form of SNP genotypes and phased haplotypes from the CEPH population (Utah residents with ancestry from northern and western Europe, CEU). The imputed data were quality controlled by excluding SNPs with a posterior probability ≤0.9, MAF <0.01, >10% missing genotypes, or with significant deviation from HWE (Exact test *P* value≤1 × 10^−10^ in cases or Exact test *P* value ≤0.001 in controls). Following this process, a total of 1,455,406 imputed SNPs remained for analysis. For the Stage-3.4 cases, the genotypes of rs9376092, rs4858647 and rs2736100 are included on the Illumina Human OmniExpressExome v1.2 platform and were therefore determined directly; rs12339666 and rs2201862 were determined by imputation.

### Statistical analysis

We used allelic *χ*^2^ to compare allele frequencies between cases and controls and calculate odds ratios (ORs) and 95% confidence intervals (CIs). To examine the possibility of confounding factors in our stage-1 data, such as population stratification and separate genotyping of cases and controls, we used the qqnorm and qqplot procedures in R^47^ to construct a quantile-quantile (Q-Q) plot of observed and expected *P* values under the null distribution (Supplementary Fig. 2). Manhattan plots were generated using HaploView 4.2 (ref. 48), and regional plots were generated using LocusZoom^49^.

The power of our stage-1 cohort to detect markers associated with the risk of developing MPNs was estimated using the genetic power calculator^12^ with values of 524 cases and 2,674 controls, and an age standardized disease prevalence of one per 5,000 European persons^50^. Modest values of genotype relative risk (1.1–2.0) were used and it was assumed that the causal allele had been tagged with *D*′=0.8 and controls are unselected. In general, study power is diminished by lower causal allele frequency, and so a range of conservative estimates (0.05–0.3) for this value were used.

GWA studies are prone to detection of false positives and our stage-1 sample size is modest. To ensure that the most promising signals were taken through to stage-2, we therefore selected SNPs on the basis of the following criteria rather than significance alone. First, a ‘clumping’ procedure^46^ was used to select 120 SNPs with allelic *χ*^2^ *P* values ≤0.001 and support from correlated SNPs (*r*^2^ ≥0.5, within 500 kb, and allelic *χ*^2^ *P* value ≤0.01). Second, 22 SNPs were selected that have allelic *χ*^2^ *P* values ≤0.02 and are within 500 kb of loci associated with normal variation in platelet number (PLT) and or platelet volume (MPV)^14^. An additional 11 SNPs with allelic *χ*^2^ *P* values ≤0.0007 were chosen on the basis of their location within or near genes with functional relevance. Finally, 50 SNPs with allelic *χ*^2^ *P* values ≤0.0002 and without correlated SNPs (*r*^2^ ≥0.5) were also selected. For regions with multiple SNPs with significant association, the most significant SNP was selected as the lead along with any other SNP(s) that were not in high LD with the lead SNP (*r*^2^ ≤0.6). Using these selection techniques a total of 203 SNPs were identified.

A fixed effects inverse variance-weighted meta-analysis of the stage-1 and stage-2 results was used to select five SNPs with meta-analysis *P* values ≤0.001 and effects in the same direction for further analysis at stage-3. The same methodology was used to determine the final effect size and significance by combining the results from the six *JAK2*^V617F^-negative cohorts. The metan module in STATA v11.0 (ref. 51) was used to perform these meta-analyses under the assumption of one true effect size (odds ratio) and that any differences in observed effects between studies are due to sampling error. Two methods were used to estimate the between-study heterogeneity, these are the *χ*^2^-based Cochran’s *Q* statistic and the *I*^2^ statistic, which describe the percentage of variation across studies that is due to heterogeneity rather than chance^52^.

To assess the impact of our findings at a population level, we computed population attributable risks (PAR), which estimate the proportion of MPN cases that can be attributed to each polymorphism. We calculated this as, PAR=(*x*−1/*x*)100, where *x*=(1−*p*)^2^+2*p*(1−*p*)OR_1_+p2OR_2_, *p* is the frequency of the risk allele in the control cohort, and OR_1_ and OR_2_ are the genotypic ORs associated with heterozygosity and homozygosity, respectively, and relative to common homozygotes^53^. A combined PAR was also calculated as PAR=1−(1−PAR_1_)(1−PAR_2_)…(1−PAR_*n*_) incorporating all risk factors.

To determine whether SNPs with significant replication were associated exclusively with *JAK2*^V617F^-negative MPNs, a Breslow–Day test was used to assess the homogeneity of effect sizes (ORs) among the V617F negative and positive cohorts (Breslow–Day *P* values <0.05 were considered statistically significant). The Breslow–Day method was also used to assess the homogeneity of effect sizes (ORs) in cases with or without somatic mutations in either *CALR* or *MPL*.

We considered the possibility that the observed associations might arise as a consequence of clonal somatic changes in the MPN cases. Although it is theoretically possible that the constitutional genotypes of the intragenic SNPs we identified might be altered by somatic point mutations, this is clearly extremely unlikely in a disorder for which the rate of somatic mutation is known to be low (median number of somatic mutations in ET and PV=6.5/exome and for MF 13/exome 3). More plausible is the possibility that constitutional genotypes might be altered by small regions of loss of heterozygosity (LOH) that are below the limit of detection of SNP arrays. LOH in the majority of cells would result in germline heterozygous SNPs being scored as homozygous. If the LOH was recurrent and occurred randomly with respect to SNP genotype then it would have no effect on the allele frequency. For example, a SNP with a MAF of 50% would normally be expected to result in a genotype distribution of 25% a/a, 50% a/b and 25% b/b. Recurrent somatic LOH in say 20% of cases would result in an apparent genotype distribution of 30% a/a, 40% a/b and 30% b/b. The MAF is still 0.5, and thus there is no effect on the association study. If, however, small deletions/aUPD were not random with respect to SNP genotype (for example, if they were part of a particular haplotype that conferred a selective advantage when lost or gained) then we would expect to see distortions in genotypes that could potentially affect the GWAS results. In the example above, if a hemizygous a/- or homozygous a/a genotype had a selective advantage over a/b, then a recurrent somatic deletion/aUPD in 20% of cases would result in an apparent genotype distribution of 35% aa, 40% ab and 25% bb. The apparent MAF is 0.45 and thus could be picked up by the GWAS if the effect was large enough. However, in both instances the genotypes would be skewed away from the expected Hardy–Weinberg equilibrium (Exact test *P* value=3.52 × 10^−6^ and exact test *P* value=1.45 × 10^−6^ for 20% LOH occurring randomly or selecting a/a genotype, respectively). All our SNPs showed close agreement with expected Hardy–Weinberg distribution: as indicated in Supplementary Table 7, none showed an exact test *P* value ≤0.05 in our data. If we simulate LOH randomly affecting just 10% of cases the exact test *P* values change markedly (final column): 5/6 deviate significantly from expected at exact test *P*≤0.05 and 3/6 deviate significantly at exact test *P*≤0.001. The lack of any significant deviation strongly suggests that clonal changes have not contributed to the significance of SNPs in this study.

### Statistical analysis of risk variants in PT-1

For continuous variables, analyses comparing diagnostic variables and genotype were performed using linear models, correcting for *JAK2* status and using the dosage of alleles as the predictor variable for the SNP of interest. For categorical variables, the analysis used the *χ*^2^-test for trend across the dosage of alleles of the SNP of interest. Clinical outcome was assessed by *χ*^2^-tests for events preceding diagnosis and log-rank test for events after trial entry. Linear mixed effect models were used to assess the relationships between the SNP and response of blood counts to therapy.

### Chromosomal abnormalities identified by analysis of SNP array data

To identify regions of acquired uniparental disomy (aUPD) and indels, PennCNV^54^ and Affymetrix Power Tools (APT www.affymetrix.com/support/developer/powertools/index.affx) were used to convert the raw CEL files into a normalized signal intensity file that were comparable in terms of raw signal intensity distributions. Log R Ratio (LRR) and B Allele Frequency (BAF) were calculated for each marker in each individual using the allele-specific signal intensities from the normalized signal intensity file. BAF segmentation^55^ was used to exclude non informative homozygous SNPs (BAF >0.9 or BAF <0.1) and SNPs where the absolute difference in BAF between preceding or succeeding SNPs was greater than 0.6. Mirrored BAF (mBAF) values were calculated by reflection at 0.5, and a circular binary segmentation (CBS) algorithm was used to identify regions of similar allelic proportions with mBAF above a threshold of 0.56. Finally, regions of allelic imbalance were compared with log R Ratios to determine whether they were indels or copy number neutral aUPD.

### Screening *JAK2*, *CALR* and *MPL* for somatic mutations

Samples were screened *JAK2*^V617F^ using a range of techniques employed at the recruiting centres. Mutations in *CALR* were detected using fragment analysis PCR and characterized using Sanger sequencing. For fragment analysis, one primer was labelled with either 6-carboxyfluorescein (6-FAM) or 6-carboxyfluorescein hexachloride (HEX). PCR was performed using 1.25U HotStar *Taq* (Qiagen, Crawley, UK), 1 x HotStar *Taq* buffer, 1.5 mM MgCl_2_, 200 μM dNTPs, 0.5 μM each primer (CALR_FA_F and CALR_FA_R, primers listed in Supplementary Table 4), 20–40 ng DNA in a 25 μl reaction. Cycling conditions were 95 °C for 10 min, 30 cycles of 95 °C for 1 min, 60 °C for 1 min, 72 °C for 1 min, followed by 72 °C for 30 min. Products were diluted between 1/500 and 1/1,000 in sterile water and resolved by capillary gel electrophoresis, by sizing on a 3130 Genomic Analyser (Life Technologies, Paisley, UK). Results were analysed using GeneMarker software version 1.85 (SoftGenetics, State College, PA, USA) and analysed on the blue (FAM) or green (HEX) channel. For Sanger sequencing, PCR was carried out using primers (CALR_seq_F and CALR_seq_R) and following identical reaction and cycling conditions to that used for fragment analysis, apart from a final extension at 72 °C for 10 min. Sequences were analysed using Mutation Surveyor 3.1 software (SoftGenetics, State College, PA, USA).

Specific pyrosequencing assays were used to detect *MPL* W515 mutations, following standard PCR, cycling and procedural methods described previously^56^. For each sample, primers designed to amplify the region in question (MPL_pyro_F and MPL_pyro_R, sequences listed in Supplementary Table 4) were used to create a biotinylated PCR product, which was then used in a pyrosequencing reaction using a sequencing primer (MPL_pyro_seq) for both the *MPL* W515L mutation and the *MPL* W515K mutation (sequences to analyse were 5′-GTKGCAGTTTCC TGCACACTACAGGTAC-3′ and 5′-GTRGCAGTTTCC TGCACACTACAGGTAC-3′, respectively). Pyrosequencing PCR products for *MPL* were also Sanger sequenced to identify rarer mutations using the reverse primer, following conditions outlined above.

We note that the frequency of *MPL* and *CALR* mutations varies between cohorts (Table 2A). Specifically, the frequency of somatic *CALR* mutations varied between 22 and 53% (average 45%), while somatic mutations in *MPL* were seen in 3 to 29% (average 11%) of cases. Concerning *MPL*, the German/Austrian cohort was not sequential and included a substantial number of *MPL*-mutated cases that were specifically collected for a previous study^10^. As for *CALR*, the mutation frequency was lower than that reported previously^2^,^3^ for all cohorts and substantially lower for the Greek cases. We believe that these differences are likely to result from the well known difficulty in accurately defining JAK2^V617F^-negative ET in routine practice by exclusion of all potential causes of reactive thrombocytosis^57^. In addition, we cannot exclude the possibility that the frequency of *CALR* mutations might vary between different MPN populations.

### Gene expression analysis

To avoid potential confounding effects of undefined somatic mutations, peripheral blood (PB) samples were obtained from anonymised healthy individuals. Mononuclear cells were cultured in methylcellulose medium (H4434; Stem Cell Technologies, Vancouver, Canada) at a density of 2 × 10^5^ cells per ml, following the manufacturer’s instructions. Colonies comprising a minimum of 100 cells were characterized based on morphology as CFU-GM, CFU-GEMM, CFU-E or BFU-E, as described by Stem Cell Technologies. For each individual, colonies were plucked and pooled according to morphology, into monocyte/granulocyte (containing CFU-GM and CFU-GEMM) and erythroid colonies (containing CFU-E and BFU-E). Cells were washed in PBS, lysed in trizol and RNA extracted and reverse transcribed into cDNA using random hexamer primers following standard procedures. *MYB* and *HBS1L* expression was measured in duplicate using TaqMan assays (Applied Biosystems) and a Corbett Rotor Gene 6000 (Corbett Life Science, Sydney, Australia).

Relative *MYB* (two alternatively spliced isoforms) and *HBS1L* expression for each target were normalized to *GUSB* expression (Supplementary Tables 4 and 5) values using the delta-delta Ct method, as described by Pfaffl *et al.*^58^ Test samples were placed into two groups according to HMIP risk allele status, either carrying one or two risk alleles (AA/AC) or no risk alleles (CC). Complementary DNA from two separate normal granulocyte samples previously defined as carrying the AC and CC risk allele genotype was used as calibrator samples to test for normality of expression distribution, and serial dilutions of cell line K562 cDNA was used to control for Q-PCR efficiency. For each sample, expression values were compared with the equivalent calibrator control, that is, the AA/AC test group was normalized to the AC calibrator control, and the CC test group was normalized to the CC calibrator control. The Mann–Whitney test was used to evaluate the difference in *MYB* and *HBS1L* expression levels, comparing normalized expression values for those that were grouped as AA/AC versus those grouped as CC, for each of the PB granulocyte, BFU-E and CFU-GM cell subtypes. Fold difference in gene expression was determined by subtracting the median normalized expression level of the CC group by that of the AA/AC group, for each cell type subset.

### Functional annotation of candidate genes

For identification of candidate genes and previous trait associations, we considered the nearest gene, and any other gene located within 500 kb of the sentinel SNP to be a candidate for mediating predisposition to MPN. Genomic regions carrying risk loci and candidate genes were investigated for other trait associations by inspecting a database of all SNP associations that reach genome-wide significance, curated by the National Human Genome Research Institute GWAS catalogue^59^. Only associations potentially relevant to haematopoiesis were considered.

To predict functional effects we used HaploReg^60^ to identify SNPs linked to the sentinel SNPs (*r*^2^>0.8 in Europeans from the 1000 Genomes Project) and, on the basis of data from the Encyclopedia of DNA Elements (ENCODE)^61^ and the NHRI Roadmap Epigenomics Mapping Consortium^62^, to annotate them with respect to (i) histone modifications, (ii) DNaseI hypersensitivity, (iii) transcription factor binding, (iv) disruption of regulatory motifs and (v) conservation across mammals estimated by GERP and SiPhy. To further investigate the sentinel and linked variants we used RegulomeDB^63^ to assign functionality scores on the basis of data from Gene Expression Omnibus (GEO), ENCODE and published literature. A RegulomeDB score of 5 and 6 indicates there is minimal evidence for functional activity. Finally, we investigated whether our index and linked SNPs overlapped with regions of nucleosome depletion generated by formaldehyde-assisted isolation of regulatory elements (FAIRE-seq), as depleted regions have been shown to have greater epigenetic activity and mark the presence of functional variants that alter the underlying DNA sequences, resulting in different binding affinities for transcription factors^64^. For this we obtained filtered and binned BED files containing FAIRE-seq data from primary human megakaryocytes and erythroblasts^65^, and intersected SNP positions with FAIRE regions to look for regions of colocalisation.

All candidate genes were investigated for expression in hematopoietic cells, by interrogating browsers of gene expression profiles generated in purified progenitors of different lineages of the human haematopoietic system (HaemAtlas^66^ and HemaExplorer^67^) and a recently published data set^68^. To investigate the relationship between the sentinel SNPs and gene expression levels, two expression quantitative trait (eQTL) browsers were used that curate derived from immortalized lymphoblastoid cell lines and primary monocytes; an eQTL browser hosted by the Pritchard laboratory^18^ and GENEVAR^69^.

## Additional information

**How to cite this article:** Tapper, W. *et al.* Genetic variation at *MECOM*, *TERT*, *JAK2* and *HBS1L-MYB* predisposes to myeloproliferative neoplasms. *Nat. Commun.* 6:6691 doi: 10.1038/ncomms7691 (2015).

## Supplementary Material

Supplementary Information Supplementary Figures 1-6, Supplementary Tables 1-7 and Supplementary References

## Figures and Tables

**Table 1:** Summary of the top SNPs showing or approaching genome-wide significant association (*P*<5 × 10^−8^) in meta-analysis.

**SNP** *	**Alleles** †	**RAF** ‡	**Gene**	***JAK2*** ^**V617F**^ **-negative cases**				***JAK2***^**V617F**^**-negative and all** ***JAK2***^**V617F**^ **positive**				***JAK2***^**V617F**^**-negative and** ***JAK2***^**V617F**^**-positive ET and MF cases**			
				***P***_***META***_	**OR (CI)**	***I*** ^**2**^	**PAR**	***P***_***META***_	**OR (CI)**	***I*** ^**2**^	**PAR**	***P***_***META***_	**OR (CI)**	***I*** ^**2**^	**PAR**
rs12339666§	T/G	0.26	*JAK2*	1.272 × 10^−10^	1.34 (1.23–1.47)	0	17.4	2.287 × 10^−62^	1.76 (1.65–1.88)	96.2	27.5	1.748 × 10^−20^	1.43 (1.33–1.55)	53.4	21.0
rs2201862	T/C	0.48	*−MECOM*	1.964 × 10^−9^	0.82 (0.77–0.88)	0	16.5	4.075 × 10^−10^	0.84 (0.80–0.89)	0	14.3	1.671 × 10^−9^	0.84 (0.79–0.89)	0	14.7
rs9376092	A/C	0.27	*HBS1L-MYB*	5.273 × 10^−6^	1.18 (1.10–1.26)	62	7.4	5.547 × 10^−7^	1.16 (1.09–1.23)	79.4	6.9	7.766 × 10^−10^	1.22 (1.14–1.30)	66.5	9.0
rs4858647	A/C	0.11	*THRB-RARB*	1.393 × 10^−5^	1.24 (1.13–1.37)	77	4.2	8.994 × 10^−5^	1.18 (1.09–1.29)	72.9	3.1	3.764 × 10^−5^	1.21 (1.10–1.32)	75.1	3.6
rs2736100‖	C/A	0.51	*TERT*	1.728 × 10^−5^	1.31 (1.16–1.47)	0	34.9	3.667 × 10^−26^	1.51 (1.40–1.63)	71.0	42.7	4.026 × 10^−13^	1.43 (1.31–1.57)	83.5	37.8

^*^rs identifier from dbSNP.

^†^Risk-associated/non-risk-associated alleles.

^‡^Frequency of risk allele in controls that were used to calculate population attributable risk (PAR). *P*_META_, fixed effects meta-analysis of allelic *χ*^2^ tests; OR, odds ratio; CI, 95% confidence interval; *I*^2^, heterogeneity index (0–100).

^§^In *JAK2*^V617F^-negative cases, meta-analysis is based on a combination of four studies instead of six. In *JAK2*^V617F^-negative and all *JAK2*^V617F^-positive cases, meta-analysis is based on a combination of six studies instead of eight. In *JAK2*^V617F^-negative and ET and PMF *JAK2*^V617F^-positive cases, meta-analysis is based on a combination of five studies instead of seven.

^‖^In *JAK2*^V617F^-negative cases, meta-analysis is based on a combination of two studies instead of six. In *JAK2*^V617F^-negative and all *JAK2*^V617F^-positive cases, meta-analysis is based on a combination of four studies instead of eight. In *JAK2*^V617F^-negative and ET and PMF *JAK2*^V617F^-positive cases, meta-analysis is based on a combination of three studies instead of seven.

**Table 2:** Frequency of somatic mutations and subtype specific effects.

**A. Frequency of somatic mutations in** ***CALR*** **and** ***MPL***				
**Cohort**	***CALR*** **No. (%)**	***MPL*** **No. (%)**	***CALR*** **and/or** ***MPL*****No. (No. double +ve)**	**Triple −ve No. (%)**
UK1	266 (51.2)	51 (9.8)	316 (1)	204 (39.2)
UK2	84 (43.3)	11 (5.7)	94 (1)	99 (51)
German & Austrian	136 (47.2)	83 (28.8)	217 (2)	69 (24)
Greek	95 (21.5)	14 (3.2)	109 (0)	333 (75.3)
UK3	168 (51.9)	29 (9)	197 (0)	127 (39.2)
Italian	322 (52.6)	56 (9.2)	377 (1)	234 (38.2)

**B. Association and comparison according to** ***JAK2***^**V617F**^ **and either** ***CALR*** **and/or** ***MPL***									
**SNP**	***JAK2***^**V617F**^**−ve** ***P***_**META**_ **(OR)**	***JAK2***^**V617F**^**+ve UK4 ET&PV** ***P***_**META**_ **(OR)**	***P***_**BD**_	***CALR*****+ve and/or*****MPL*****+ve** ***P***_**META**_ **(OR)**	**Triple −ve** ***P***_**META**_ **(OR)**	***P***_**BD**_ **CALR+ve and/or MPL+ve versus triple −ve**	***JAK2***^**V617F**^**+ve UK4 ET** ***P***_**META**_ **(OR)**	***JAK2***^**V617F**^**+ve UK4 PV** ***P***_**META**_ **(OR)**	***P***_**BD**_ ***JAK2***^**V617F**^**+ve ET versus** ***JAK2***^**V617F**^**+ve PV**
rs12339666	1.272 × 10^−10^ (1.34)	5.43 × 10^−62^ (2.43)	2.82 × 10^−18^	1.299 × 10^−8^ (1.40)	0.0002 (1.28)	0.0266	5.344 × 10^−13^ (1.68)	1.286 × 10^−75^ (3.50)	2.452 × 10^−11^
rs2736100	1.728 × 10^−5^ (1.31)	1.92 × 10^−21^ (1.66)	0.0026	0.0003 (1.32)	0.0179 (1.26)	0.6555	8.179 × 10^−12^ (1.62)	3.153 × 10^−14^ (1.71)	0.3864
rs2201862	1.964 × 10^−9^ (0.82)	0.0368 (0.89)	0.3218	2.628 × 10^−6^ (0.82)	5.08 × 10^−5^ (0.83)	0.2237	0.1716 (0.91)	0.0733 (0.88)	0.9684
rs9376092	5.273 × 10^−6^ (1.18)	0.0566 (1.12)	0.4967	3.869 × 10^−6^ (1.24)	0.0493 (1.11)	4.487 × 10^−7^	2.62 × 10^−6^ (1.41)	0.0683 (0.87)	0.0004
rs4858647	1.393 × 10^−5^ (1.24)	0.6891 (1.04)	0.0146	0.0010 (1.23)	0.0072 (1.21)	0.1018	0.7167 (1.04)	0.8049 (1.03)	0.4167

(A) Number and percentage of cases with somatic mutations in *CALR* and *MPL* in each population. Cases with somatic mutations in *CALR* and *MPL* are described as double +ve. Triple −ve refers to cases without somatic mutation in *JAK2*, *CALR* and *MPL*. In cohorts with double positives, the percentage of cases will sum to just over 100. (B) *P*_META_ and odds ratio (OR) from fixed effects meta-analyses in subsets of cases defined by the somatic status of *JAK2*, *CALR* and *MPL*. *P*_BD_, Breslow–Day test *P* value.

**Table 3:** Comparison between ET and PV subtype in *JAK2* ^V617F^ positive cases.

**SNP**	**Gene**	**Alleles**	**PV MAF**	**ET MAF**	***P*** **value**	**OR (CI)**
rs12339666	*JAK2*	T/G	0.5627	0.3815	4.788 × 10^−16^	2.09 (1.75–2.49)
rs2201862	*−MECOM*	T/C	0.4817	0.489	0.7523	0.97 (0.81–1.17)
rs9376092	*HBS1L-MYB*	A/C	0.2495	0.3507	7.475 × 10^−7^	0.62 (0.51–0.75)
rs4858647	*THRB-RARB*	A/C	0.1000	0.1012	0.9286	0.99 (0.74–1.32)
rs2736100	*TERT*	C/A	0.6426	0.6303	0.5663	1.06 (0.88–1.27)

*P*-value, odds ratio (OR), and 95% confidence interval (CI) for comparison of JAK2^V617F^-positive PV (*n*=505) and ET cases (*n*=499) using allelic *χ*^2^-test.
