# Supplementary material for: Genetic variation at MECOM, TERT, JAK2 and HBS1L-MYB predisposes to myeloproliferative neoplasms
Source: Nat Commun. 2015 Apr 7;6:6691. doi: 10.1038/ncomms7691 (PMC4396373; doi:10.1038/ncomms7691)
Supplement: Supplementary Information — Supplementary Figures 1-6, Supplementary Tables 1-7 and Supplementary References [file ncomms7691-s1.pdf]

Supplementary information

Supplementary figure 1. Flow chart of study design

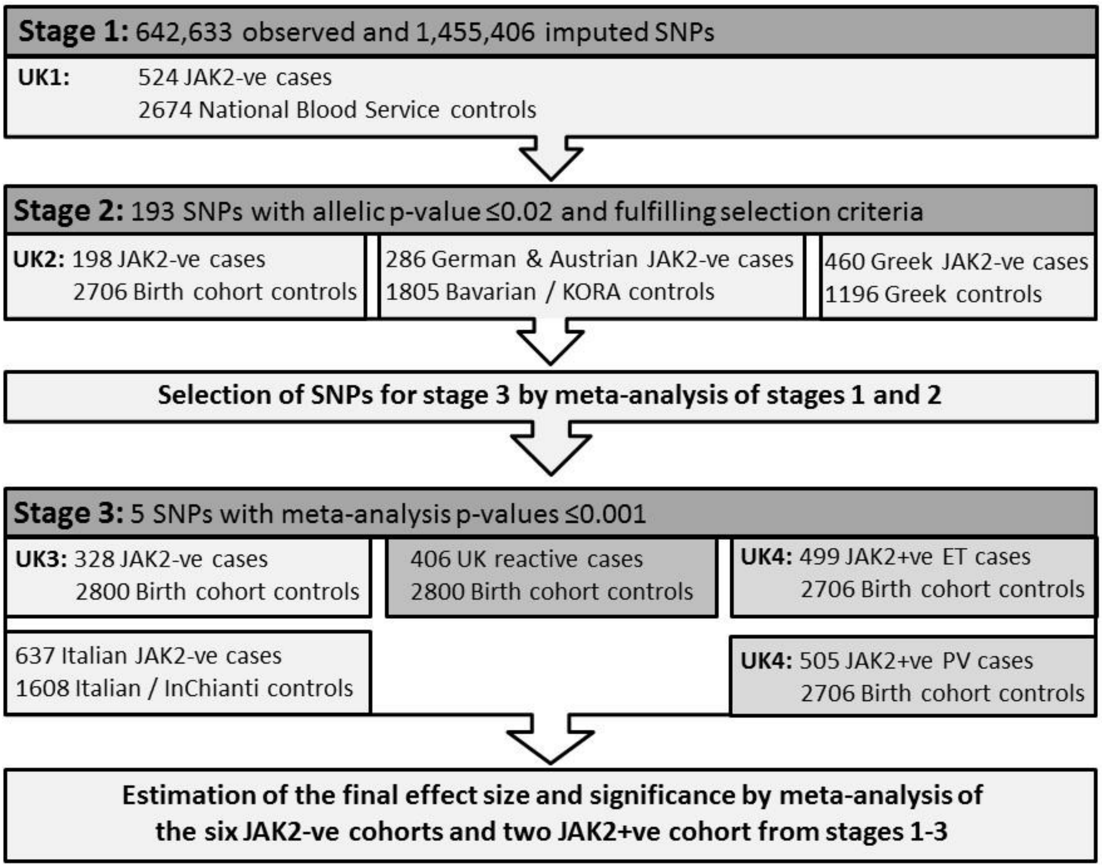

Supplementary Figure 2. Quantile-quantile plot of stage 1 results

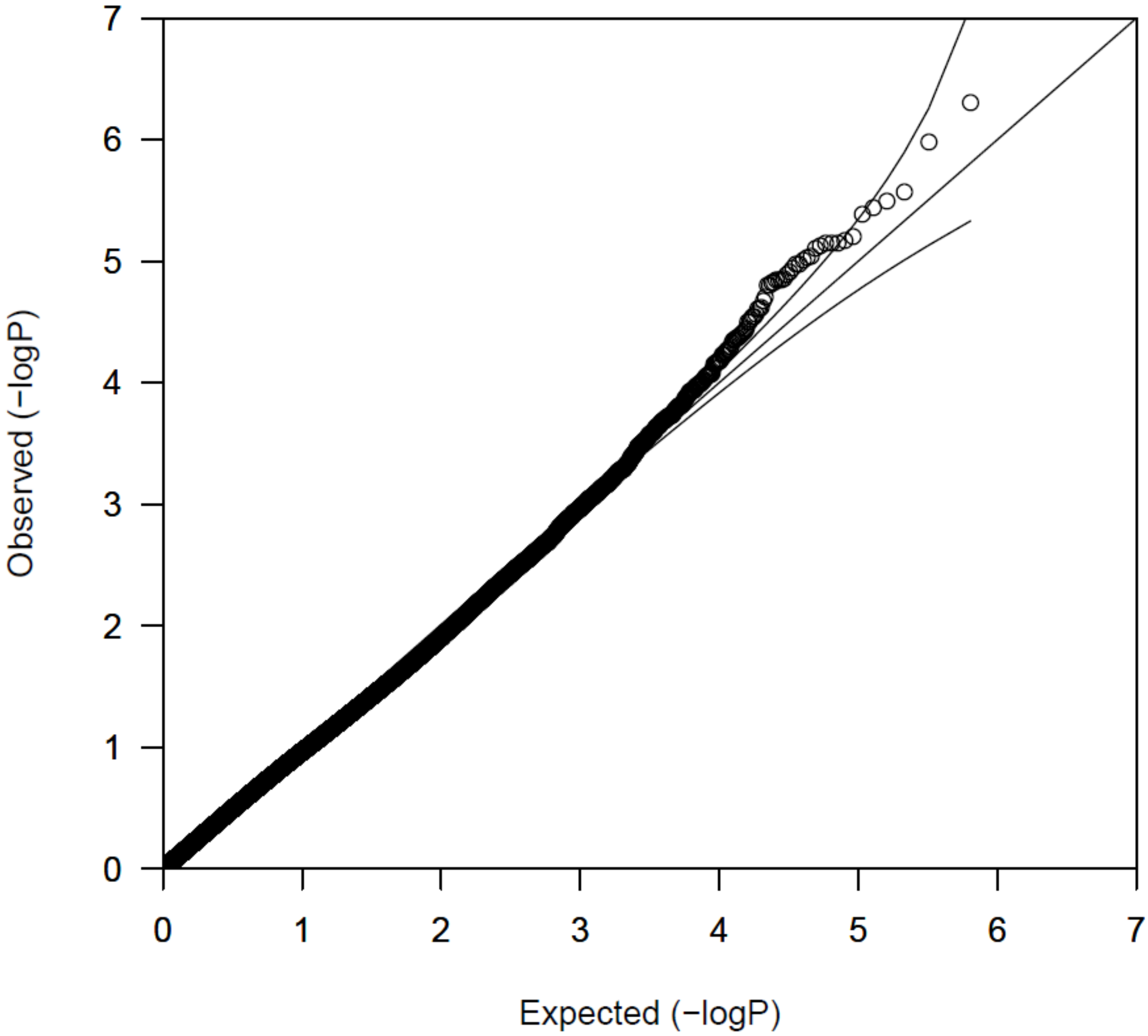

QQ plot of the observed -log10 P-values (y axis) versus the expected -log10 rank P-values (x axis) for allelic chi-square tests of association of 642,633 SNPs, with the risk of developing *JAK2*^{V617F} negative MPN (genomic inflation factor, λ=1) in the discovery phase. The region between the curved lines indicates bootstrapped 95% confidence intervals. The diagonal black line indicates expected results under null hypothesis.

Supplementary Figure 3. A plot of the stage 1 GWAs in the region of *CALR* showing no evidence for germline variation contributing to the acquisition of somatic *CALR* mutations

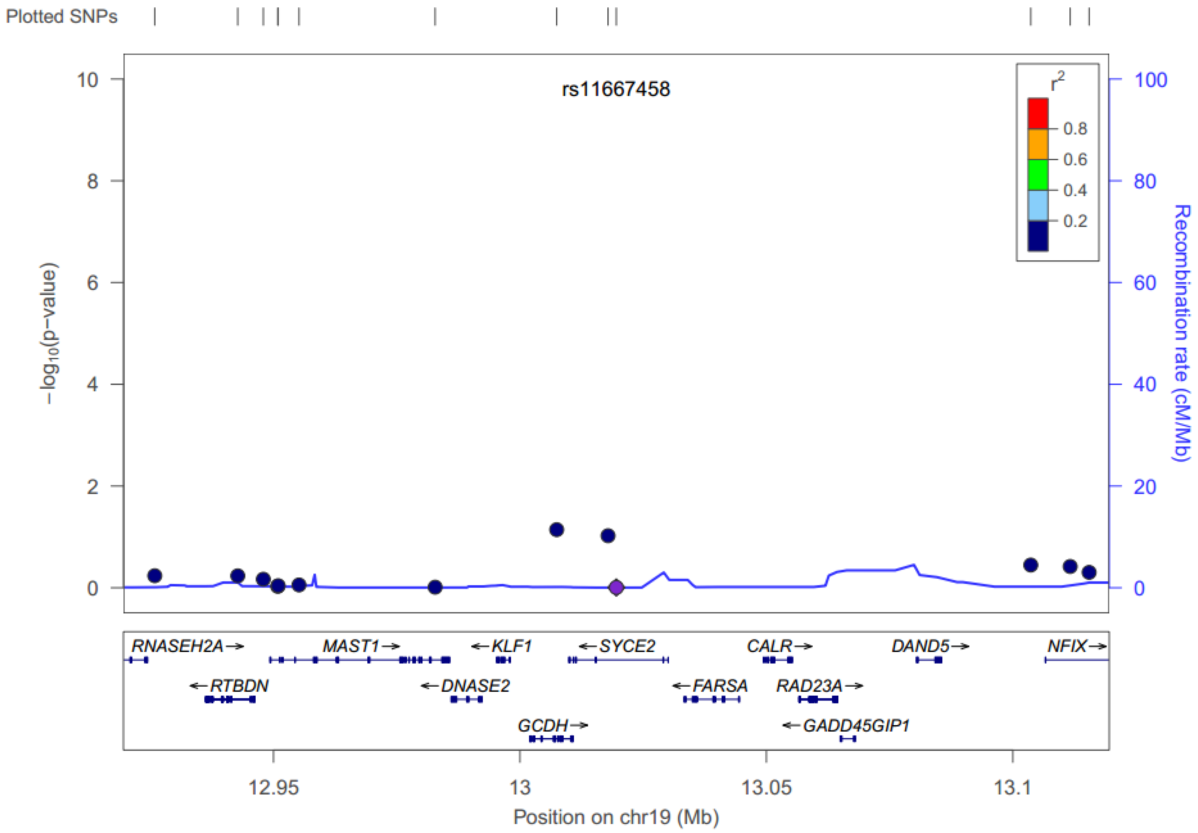

Supplementary Figure 4.

A) MYB **(two alternatively spliced isoforms represented by exons 5-6 and exons 10-13) and *HBS1L* expression quantified in normal individuals with one or two *HMIP* risk alleles (rs9376092: AA or AC) compared to those with no risk allele (rs9376092: CC) performed on A) PB granulocytes, B) erythroid progenitors (BFU-E), and C) granulocyte-macrophage progenitors (CFU-GM).**

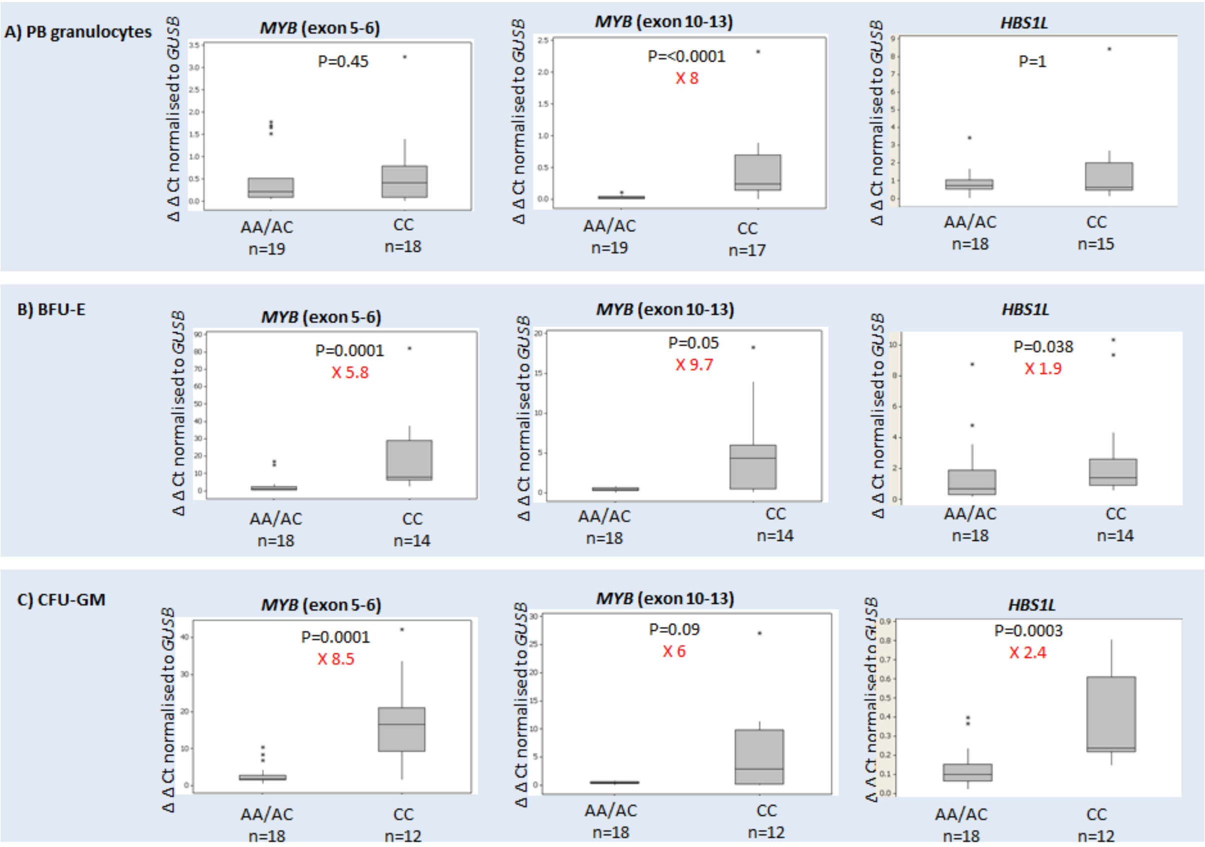

MYB and *HBS1L* expression was normalised to *GUSB*, and after grouping samples according to rs9376092 genotype (only one case was AA and so AA and AC genotypes were combined), expression levels were compared using the Mann-Whitney test. The fold difference in median expression is indicated in red.

B). Expression analysis in BFU-E colonies of MPN cases (PV, *n*=16; ET, *n*=20). Individual colonies were plucked, genotyped, pooled and analysed by Illumina Human-6 v2.0 Expression BeadChips as previously described [1]. Results of wild type (WT) and *JAK2*^{V617F} mutant (VF) colonies from individual cases are shown in pale blue for ET and pale red for PV. The mean for each subtype is indicated by the strong blue and red lines and shows a significant difference for *MYB* expression in ET (Mann-Whitney test ***P***= 0.0009; q=0.01) but no other significant differences.**

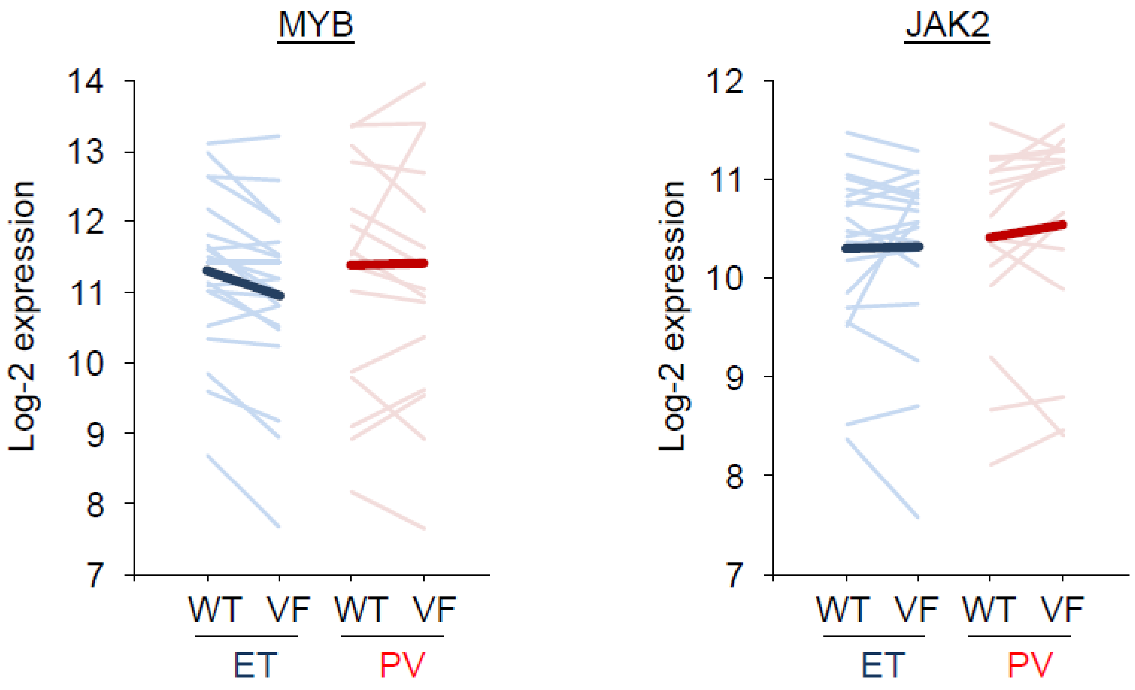

Supplementary Figure 5. Copy number changes and regions of acquired uniparental disomy (aUPD) in the 524 Stage 1 cases

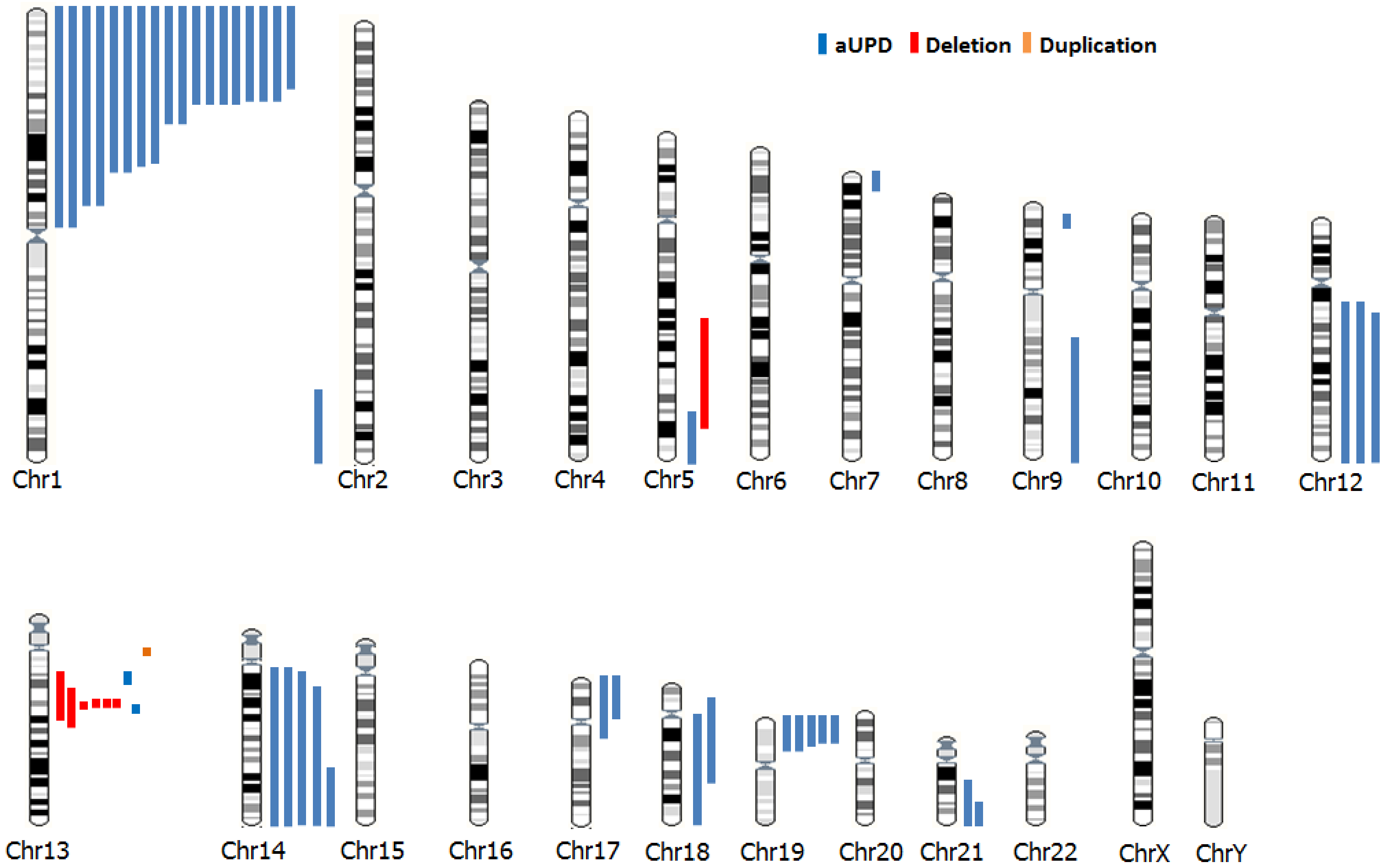

Selected cases were investigated for somatic mutations: of the 18 cases with 1p aUPD, 11/17 tested had *MPL* W515 mutations; the single case with 9p aUPD tested negative for *JAK2* exon12 and 14 mutations; 5/5 cases with 19p aUPD had *CALR* mutations, of which 2 were Type 1 and 3 were Type 2 [2]

Supplementary Figure 6. Multi-dimensional Scaling (MDS) plot of ethnicity clustering

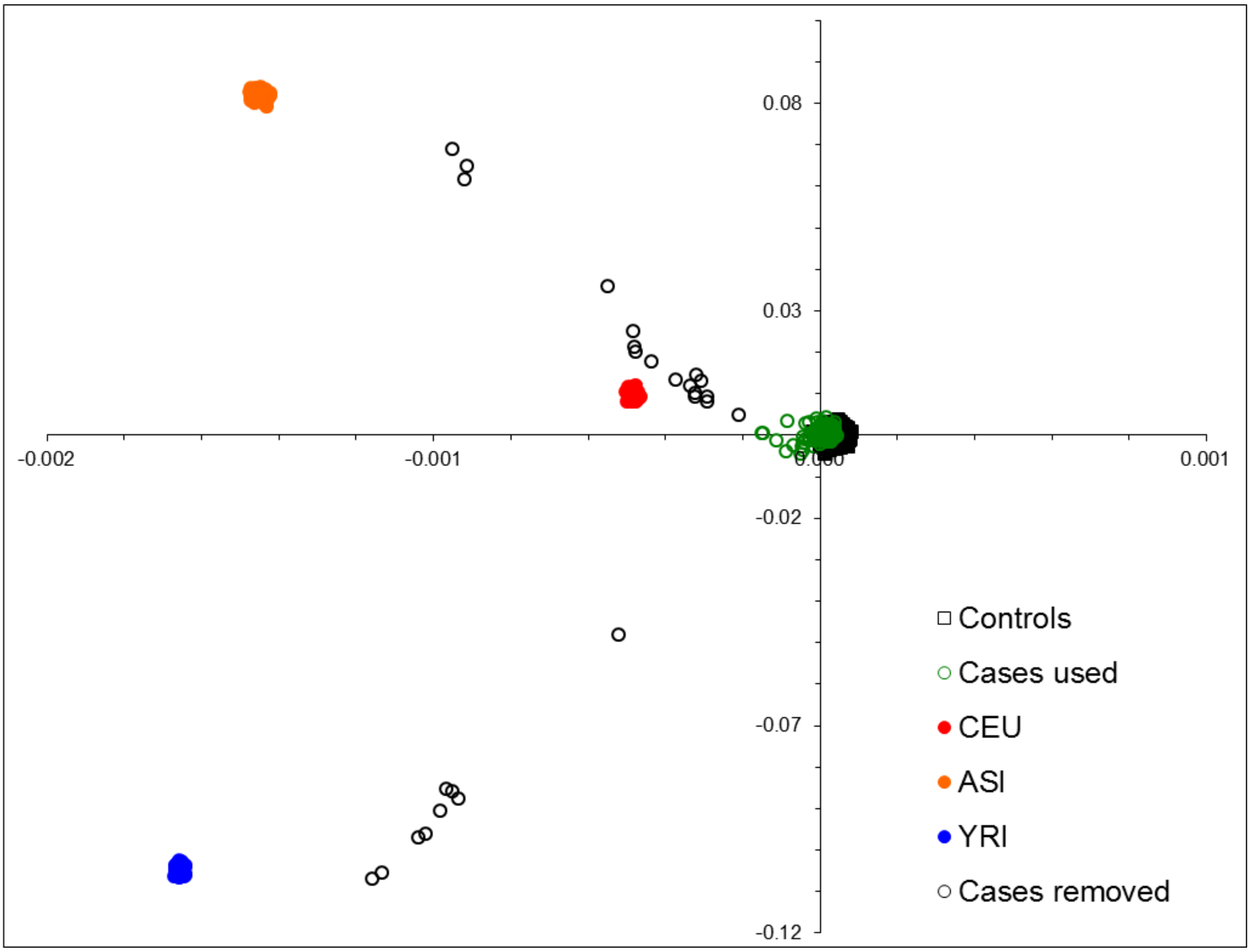

MPN cases (green and black circles) and WTCCC2 controls (black squares) are plotted along with data from HapMap for Utah residents with ancestry from northern and western Europe (CEU, red circles), Japanese and Han Chinese individuals from Tokyo and China (ASI, orange circles), Yoruban individuals from Ibadan, Nigeria (YRI, blue circles). Ethnic groups form separate clusters and MPN cases and WTCCC2 controls cluster together which indicates that they are suitable for comparison. The black circles are MPN cases (*n*=26) that were removed from the analysis because their genotypes did not concur with a European ancestry.

Supplementary Table 1. Unadjusted p-values for lead SNPs with replication (rsids in bold), independent SNPs from the same region (unformatted rsids) and published SNPs (rsid in italics)

JAK2 status	*JAK2*^{V617F} negative						*JAK2*^{V617F} positive			NA
**Stage & SNPs**	**Stage 1 (no. of SNPs=2098039)**	**Stage 2 (no. of SNP =193)**			**Stage 3 (no. of SNPs=5)**		**Stage 3 (no. of SNPs=6)**			**Stage 3 (no. of SNPs =5)**
**Cases**	**UK1 (*n*=524)**	**UK2 (*n*=198)**	**German (*n*=187) Austrian (*n*=99)**	**Greek (*n*=460)**	**UK3 (*n*=328)**	**Italian (*n*=637)**	**UK4 ET and PV (*n*=1004)**	**UK4 ET only (*n*=499)**	**UK4 PV only (*n*=505)**	**UK reactive (*n*=406)**
**Controls**	**UK national blood service (*n*=2674)**	**UK British birth cohort (*n*=2706)**	**Bavarian (KORA, *n*=1805)**	**Greek (*n*=1196)**	**UK British birth cohort & UK (*n*=2800)**	**Italian (INCHIANTI, *n*=1608)**	**UK British birth cohort (*n*=2706)**	**UK British birth cohort (*n*=2706)**	**UK British birth cohort (*n*=2706)**	**UK British birth cohort & UK (*n*=2800)**
**SNP**	***P* (OR)**	***P* (OR)**	***P* (OR)**	***P* (OR)**	***P* (OR)**	***P* (OR)**	***P* (OR)**	***P* (OR)**	***P* (OR)**	***P* (OR)**
**rs12339666**	0.000605 (1.29)	**0.00025*† (1.49)**	**0.00027*† (1.41)**	**0.02457 (1.26)**			**5.43x10-62*† (2.43)**	**5.344x10^{-13}*† (1.68)**	**1.286x10^{-75}*† (3.50)**	
**rs2201862**	0.001301 (0.80)	**0.00028*† (0.68)**	0.1611 (0.88)	0.07322 (0.87)	**0.03847 (0.84)**	**0.00631† (0.83)**	**0.03681 (0.89)**	0.1716 (0.91)	0.07326 (0.88)	0.1497 (0.89)
rs4955627	0.000304 (1.28)	**0.03847 (1.25)**	0.7278 (1.03)	0.6185 (0.95)						
rs16853092	1.48x10^{-5} (1.55)	**0.02134 (1.43)**		0.8993 (0.98)						
**rs9376092**	0.001619 (1.26)	0.7106 (1.04)	**0.00078† (1.39)**	0.3903 (0.92)	0.3182 (1.10)	**0.00099*† (1.28)**	0.05655 (1.12)	**2.62x10^{-6}*† (1.41)**	*0.06825 (0.87)*	0.3854 (1.08)
**rs4858647**	0.000587 (1.42)	0.5773 (1.10)	**0.00167† (1.57)**	**0.0179 (1.29)**	**0.00366*† (1.43)**	*0.02232 (0.75)*	0.6891 (1.04)	0.7167 (1.04)	0.8049 (1.03)	0.3009 (1.14)
rs4858594	0.000539 (0.79)	0.6073 (1.06)	0.5363 (1.06)	0.4426 (1.07)						
rs4858576	0.000148 (0.71)	0.1022 (0.80)	0.5497 (1.07)	0.6037 (0.94)						
rs4858095	4.006x10^{-6} (0.68)	0.5813 (1.07)	0.944 (0.99)	0.6829 (0.96)						
**rs2736100**	0.000409 (1.27)		0.00830 (1.49)				**1.92x10^{-21}*† (1.66)**	**8.179x10^{-12}*† (1.62)**	**3.153x10^{-14}*† (1.71)**	

Abbreviation: *P* = Unadjusted *P*-value for allelic chi-square test. OR = Estimated odds ratio. *Significant after Bonferroni correction for 193 tests at stage 2 and 5 tests at stage 3. †Significant after correction for False Discovery Rate. Nominally significant (*P*-value ≤0.05) replication in bold. Values in italics indicate effects in opposite direction compared with stage 1. Cells are blank if the SNP was not genotyped.

Supplementary Table 2. ***MYB*** **and** ***HBS1L*** **expression data in healthy controls**

Gene	cell type	Genotype group	N	median normalised expression level (range)	*P* value	fold expression decrease (median normalised expression level of AA/AC group/CC group)
MYB (exons 5-6)	PB granulocytes	AA/AC	19	0.22 (0.04-1.78)	0.4566	n/s
		CC	18	0.42 (0-3.24)		
	BFU-E	AA/AC	18	1.33 (0.37-17)	0.0001	5.8
		CC	14	7.76 (2.38-82.2)		
	CFU-GM	AA/AC	18	1.93 (0.54-10.4)	0.0001	8.5
		CC	12	16.5 (1.66-42.2)		
MYB (exons 10-13)	PB granulocytes	AA/AC	19	0.03 (0-0.11)	<0.0001	8
		CC	17	0.24 (0-2.33)		
	BFU-E	AA/AC	18	0.445 (0.02-0.85)	0.0056	9.7
		CC	14	4.31 (0.07-18.3)		
	CFU-GM	AA/AC	18	0.485 (0.05-0.84)	0.0945	6
		CC	12	2.92 (0.03-27.1)		
HBS1L	PB granulocytes	AA/AC	18	0.72 (0.01-3.43)	1	n/s
		CC	15	0.62 (0.11-8.42)		
	BFU-E	AA/AC	18	0.715 (0.18-8.75)	0.0384	1.95
		CC	14	1.4 (0.59-10.3)		
	CFU-GM	AA/AC	18	0.1 (0.02-0.4)	0.0003	2.4
		CC	12	0.24 (0.15-0.81)		

Supplementary Table 3. Laboratory and clinical features at diagnosis and major thrombotic, hemorrhagic and transformation events after trial entry and in the year before diagnosis, in *JAK2*^{V617F} negative patients compared to MPN risk allele genotype.

A. rs2201862

	homozygous minor allele	heterozygous	homozygous major allele	*P* value[1]
**Genotypes of *JAK2* V617F negative MPN (*n*=308)**				
rs2201862 genotypes	CC	CT	TT	
Number (frequency)	56 (0.182)	145 (0.471)	107 (0.347)	
**Laboratory and clinical features at diagnosis**				
Age (yr.)- mean ± SD	52 ± 16	53 ± 16	52 ± 15	0.281
Hemoglobin (g/L)- mean ± SD	13 ± 1.3	14 ± 1.4	14 ± 1.2	0.143
White cells (x10*9/L)- mean ± SD	9.2 ± 2.6	9.4 ± 2.8	9.5 ± 2.6	0.515
Platelet count (x10*9/L)- mean ± SD	1060 ± 377	1040 ± 339	981 ± 319	0.101
**Major thrombotic, hemorrhagic and transformation events after trial entry and in year before diagnosis**				
**Arterial thrombosis**				
In year before diagnosis	18	29	12	0.970
After trial entry	14	17	10	0.594
**Venous thromboembolism**				
In year before diagnosis	6	3	3	0.470
After trial entry	6	6	3	0.799
**Major haemorrhage**				
After trial entry	6	20	4	0.112
**Death**	13	28	12	0.374
**Hematological transformation**				
Myelofibrosis	9	6	5	0.302
MDS/AML	2	4	2	0.875
Polycythemia vera	3	3	0	0.422
**Response to therapy**				
Effect of SNP on response to therapy for Hb				0.150
Effect of SNP on response to therapy for Plt count				0.846
Effect of SNP on response to therapy for WCC				0.808

B. rs9376092

	homozygous minor allele	heterozygous	homozygous major allele	*P* value[1]
**Genotypes of *JAK2* V617F negative MPN (*n*=308)**				
rs9376092 genotypes	AA	AC	CC	
Number (frequency)	27 (0.088)	145 (0.471)	136 (0.442)	
**Laboratory and clinical features at diagnosis**				
Age (yr.)- mean ± SD	55 ± 17	53 ± 16	51 ± 15	0.722
Hemoglobin (g/L)- mean ± SD	13 ± 1.2	13 ± 1.4	14 ± 1.3	0.212
White cells (x10*9/L)- mean ± SD	9.2 ± 2.5	9.2 ± 2.6	9.5 ± 2.8	0.500
Platelet count (x10*9/L)- mean ± SD	978 ± 269	1030 ± 345	1050 ± 368	0.548
**Major thrombotic, hemorrhagic and transformation events after trial entry and in year before diagnosis**				
**Arterial thrombosis**				
In year before diagnosis	7	29	22	0.430
After trial entry	7	19	15	0.353
**Venous thromboembolism**				
In year before diagnosis	3	7	2	0.033
After trial entry	4	5	6	0.150
**Major haemorrhage**				
After trial entry	7	11	11	0.073
**Death**	5	26	22	0.808
**Hematological transformation**				
Myelofibrosis	1	11	8	0.529
MDS/AML	0	5	2	0.301
Polycythemia vera	0	2	4	0.473
**Response to therapy**				
Effect of SNP on response to therapy for Hb				0.858
Effect of SNP on response to therapy for Plt count				0.100
Effect of SNP on response to therapy for WCC				0.731

C. rs4858647

	homozygous minor allele	heterozygous	homozygous major allele	*P* value[1]
**Genotypes of *JAK2* V617F negative MPN (*n*=306)**				
rs4858647 genotypes	AA	AC	CC	
Number (frequency)	7 (0.023)	73 (0.239)	226 (0.739)	
**Laboratory and clinical features at diagnosis**				
Age (yr.)- mean ± SD	52 ± 22	50 ± 16	53 ± 16	0.583
Hemoglobin (g/L)- mean ± SD	13 ± 1.2	14 ± 1.3	14 ± 1.3	0.769
White cells (x10*9/L)- mean ± SD	9.1 ± 2.3	9.4 ± 2.6	9.4 ± 2.7	0.566
Platelet count (x10*9/L)- mean ± SD	962 ± 181	1030 ± 361	1040 ± 348	0.597
**Major thrombotic, hemorrhagic and transformation events after trial entry and in year before diagnosis**				
**Arterial thrombosis**				
In year before diagnosis	1	10	48	0.390
After trial entry	2	6	33	0.187
**Venous thromboembolism**				
In year before diagnosis	0	3	9	0.970
After trial entry	0	0	15	0.110
**Major haemorrhage**				
After trial entry	1	4	25	0.486
**Death**	3	11	39	0.052
**Hematological transformation**				
Myelofibrosis	0	7	13	0.212
MDS/AML	0	2	5	0.823
Polycythemia vera	0	2	4	0.745
**Response to therapy**				
Effect of SNP on response to therapy for Hb				0.023
Effect of SNP on response to therapy for Plt count				0.686
Effect of SNP on response to therapy for WCC				0.912

D. rs17049659

	homozygous minor allele	heterozygous	homozygous major allele	*P* value[1]
**Genotypes of *JAK2* V617F negative MPN (*n*=304)**				
rs17049659 genotypes	CC	CT	TT	
Number (frequency)	0 (0)	23 (0.076)	281 (0.924)	
**Laboratory and clinical features at diagnosis**				
Age (yr.)- mean ± SD	0	57 ± 18	52 ± 16	0.695
Hemoglobin (g/L)- mean ± SD	0	13 ± 1.5	14 ± 1.3	0.321
White cells (x10*9/L)- mean ± SD	0	9.1 ± 2.6	9.4 ± 2.7	0.875
Platelet count (x10*9/L)- mean ± SD	0	1110 ± 478	1030 ± 333	0.921
**Major thrombotic, hemorrhagic and transformation events after trial entry and in year before diagnosis**				
**Arterial thrombosis**				
In year before diagnosis	0	2	57	0.370
After trial entry	0	4	37	0.242
**Venous thromboembolism**				
In year before diagnosis	0	2	10	0.120
After trial entry	0	1	12	0.654
**Major haemorrhage**				
After trial entry	0	2	28	0.718
**Death**	0	4	48	0.427
**Hematological transformation**				
Myelofibrosis	0	1	19	0.991
MDS/AML	0	0	7	0.548
Polycythemia vera	0	1	5	0.239
**Response to therapy**				
Effect of SNP on response to therapy for Hb				0.287
Effect of SNP on response to therapy for Plt count				0.749
Effect of SNP on response to therapy for WCC				0.381

[1]For response to therapy, this was assessed by chi-squared test for trend for events preceding diagnosis and log-rank test for events after trial entry.

Supplementary Table 4. Primer sequences

Gene	Primer name	Sequence (5’-3’)
GUSB	GUSB_EAC_F	GAAAATATGTGGTTGGAGAGCTCATT
	GUSB_EAC_R	CCAGCACTCTCGTCGGTGACTGTTCA
	GUSB_EAC_probe	6-FAM-CCGAGTGAAGATCCCCTTTTTA–BHQ1
HMIP	rs9376092seq_F	AAATGGCAGCTGGTGAAGTCAGAA
	rs9376092seq_R	GAAGAGTGGTGATCATTGGGTTGG
CALR	CALR seq_F	CTGGTCCTGGTCCTGATGTC
	CALR_seq_R	CAGTCCAGCCCTGGAGGCAG
CALR	CALR_FA_F	GCAGCAGAGAAACAAATGAAGGACA
	CALR_FA_R	CCTCATCCTCATCTTTGTCCTCATCA
MPL	MPL_pyro_F	TGACCGCTCTGCATCTAGTG
	MPL_pyro_R	Bio-GGGTCACAGAGCGAACCA
	MPL_pyro_seq	CCTGCTGCTGCTGAG

Supplementary Table 5. TaqMan assays

Gene	Exon location	ABI assay number
MYB	5-6	Hs00920558_m1
	10-11, 11-12, 12-13	Hs00920563_g1
HBS1L	1-2	Hs04188641_g1

Supplementary Table 6. Association after stage 1 and meta-analysis including and excluding cases with LOH

	Stage 1				Meta analysis			
	Including LOH		Excluding LOH		Including LOH		Excluding LOH	
SNP	*p*	OR	*P*	OR	*P*	OR	*P*	OR
rs12339666	0.000605	1.29	0.0003712	1.31	$1.27x10^{-10}$	1.34	$7.19x10^{-11}$	1.35
rs2201862	0.001301	0.80	0.001813	0.80	$1.96x10^{-9}$	0.82	$2.68x10^{-9}$	0.82
rs9376092	0.001619	1.26	0.00332	1.25	$5.27x10^{-6}$	1.18	$9.28x10^{-6}$	1.17
rs4858647	0.000587	1.42	0.01667	1.30	$1.39x10^{-5}$	1.24	0.0001	1.22
rs2736100	0.000409	1.27	0.0003678	1.29	$1.73x10^{-5}$	1.31	$1.51x10^{-5}$	1.32

Supplementary Table 7. Exact test for deviation from Hardy-Weinberg equilibrium in MPN cases

	Heterozygosity in actual data			Heterozygosity after simulation of LOH randomly affecting 10% of cases		
SNP	Observed	Expected	*P*	Observed	Expected	*P*
rs12339666	0.4398	0.4247	0.4712	0.3327	0.4025	0.0001264
rs2201862	0.4905	0.4949	0.8599	0.4447	0.5	0.01135
rs9376092	0.437	0.439	0.9208	0.3779	0.4197	0.02237
rs4858647	0.2226	0.2326	0.3441	0.1631	0.2396	1.524x10-10
rs2736100	0.521	0.4913	0.1829	0.4275	0.4999	0.0009014

Supplementary References

1. Chen, E., *et al.* Distinct clinical phenotypes associated with JAK2^{V617F} reflect differential JAK2 signalling. *Cancer Cell*. 18, 524-535 (2010)
2. Klampfl, T. *et al*. Somatic mutations of calreticulin in myeloproliferative neoplasms. *N Engl J Med*. **369**, 2379-2390 (2013).
